# Variation in supplemental lighting quality influences key aroma volatiles in hydroponically grown ‘Italian Large Leaf’ basil

**DOI:** 10.3389/fpls.2023.1184664

**Published:** 2023-06-20

**Authors:** Hunter A. Hammock, Carl E. Sams

**Affiliations:** Department of Plant Sciences, The University of Tennessee, Knoxville, TN, United States

**Keywords:** controlled environment agriculture, light-emitting diodes, narrowband LEDs, spectral quality, *Ocimum basilicum*, supplemental lighting, secondary metabolism, terpenes

## Abstract

The spectral quality of supplemental greenhouse lighting can directly influence aroma volatiles and secondary metabolic resource allocation (i.e., specific compounds and classes of compounds). Research is needed to determine species-specific secondary metabolic responses to supplemental lighting (SL) sources with an emphasis on variations in spectral quality. The primary objective of this experiment was to determine the impact of supplemental narrowband blue (B) and red (R) LED lighting ratios and discrete wavelengths on flavor volatiles in hydroponic basil (*Ocimum basilicum* var. Italian Large Leaf). A natural light (NL) control and different broadband lighting sources were also evaluated to establish the impact of adding discrete and broadband supplements to the ambient solar spectrum. Each SL treatment provided 8.64 mol^**.**^m^-2**.**^d^-1^ (100 µmol^**.**^m^-2**.**^s^-1^, 24 h^**.**^d^-1^) photon flux. The daily light integral (DLI) of the NL control averaged 11.75 mol^**.**^m^-2.^d^-1^ during the growth period (ranging from 4 to 20 mol^**.**^m^-2.^d^-1^). Basil plants were harvested 45 d after seeding. Using GC-MS, we explored, identified, and quantified several important volatile organic compounds (VOCs) with known influence on sensory perception and/or plant physiological processes of sweet basil. We found that the spectral quality from SL sources, in addition to changes in the spectra and DLI of ambient sunlight across growing seasons, directly influence basil aroma volatile concentrations. Further, we found that specific ratios of narrowband B/R wavelengths, combinations of discrete narrowband wavelengths, and broadband wavelengths directly and differentially influence the overall aroma profile as well as specific compounds. Based on the results of this study, we recommend supplemental 450 and 660 nm (± 20 nm) wavelengths at a ratio of approximately 10B/90R at 100-200 µmol^**.**^m^-2.^s^-1^, 12-24 h^**.**^d^-1^ for sweet basil grown under standard greenhouse conditions, with direct consideration of the natural solar spectrum and DLI provided for any given location and growing season. This experiment demonstrates the ability to use discrete narrowband wavelengths to augment the natural solar spectrum to provide an optimal light environment across variable growing seasons. Future experiments should investigate SL spectral quality for the optimization of sensory compounds in other high-value specialty crops.

## Introduction

Light plays a crucial role in the growth, yield, and metabolic processes of plants (Schmitt and Wulff, 1993; Massa et al., 2008; Olle and Viršile, 2013). It is one of the most important abiotic factors that regulate various physiological signals as well as primary (Darko et al., 2014; Thoma et al., 2020) and secondary (Ouzounis et al., 2015; Landi et al., 2020) metabolic responses in plants. The quality, intensity, and photoperiod of light all directly impact plant growth and development (Smith, 1982; Fausey et al., 2005; Jiao et al., 2007). Photosynthetically active radiation (PAR) is composed of different wavelengths within the visible spectrum (400-700 nm) (McCree, 1973), but ultraviolet (Behn et al., 2010; Sakalauskaite et al., 2013; Santin et al., 2021) and far-red (Halaban, 1969; Mokvist et al., 2014; Kalaitzoglou et al., 2019; Zhen and Bugbee, 2020) wavelengths can be perceived and utilized by many species of higher plants.

The roles of light in activating pathways that shape plant growth and development are multifaceted and complex (Fankhauser and Chory, 1997; Chen et al., 2004). Plants possess a unique array of photoreceptors that sense various wavebands across the spectrum (Fankhauser and Chory, 1997; Folta and Carvalho, 2015; Galvao and Fankhauser, 2015). These include phytochromes which detect red and far-red light, cryptochromes which detect ultraviolet, blue, and green light; and phototropins, which respond primarily to blue light (Casal, 2000; Briggs and Olney, 2001). These sensors initiate downstream physiological and metabolic changes (Chen et al., 2004; Casal and Yanovsky, 2005; Rockwell et al., 2006). For example, isoprenoid and phenylpropanoid synthesis are differentially affected by the spectral quality of light received (Bourgaud et al., 2001; Vranova et al., 2012; Lu et al., 2017). Overlapping interactions between narrowband wavelengths have been shown to cause synergistic or antagonistic effects on primary and secondary metabolic pathways (Colquhoun et al., 2013; Carvalho et al., 2016; Pennisi et al., 2019a).

Discrete narrowband wavelengths within the natural solar spectrum are known to play an important role in the quality of plants, affecting flavor, aroma, color, texture, and other human sensory aspects (Kelly and Runkle, 2020; Hammock et al., 2021; Paradiso and Proietti, 2021). Altering the spectral quality of light provided to greenhouse crops, whether that be using filters or supplemental lighting (SL), can directly influence secondary metabolic pathways (Ouzounis et al., 2015; Pennisi et al., 2019a). Different wavelengths of light have been shown to produce other effects in high-value specialty crops such as herbs (Dou et al., 2017; Pennisi et al., 2019b; Larsen et al., 2020), spices (Dou et al., 2017), flowers (Colquhoun et al., 2010; Currey et al., 2012; Colquhoun et al., 2013), strawberries (Kasperbauer et al., 2001), tomatoes (Gómez and Mitchell, 2014; Kaiser et al., 2018; Dannehl et al., 2021), and tea leaves (Fu et al., 2015); all of these crops could utilize variable light exposure to modify volatile metabolites responsible for their sensory qualities (Carvalho et al., 2016). For example, narrowband red and blue wavelengths are known to improve the sensory quality of certain crops. Manipulating the spectral quality of greenhouse crops using SL has been shown to enhance the production of secondary metabolites, which can be used for culinary, medicinal, and commercial purposes (Holopainen et al., 2018).

Horticultural lighting systems are sometimes employed in greenhouse operations when natural light intensity and/or spectral quality are insufficient for sustained plant growth and development (Faust et al., 2005; Sipos et al., 2020). It is well known that solar spectral quality, irradiance, daily light integral (DLI), and photoperiod are variable depending on the time of day, the year, location, and local weather patterns (Korczynski et al., 2002; Thorne et al., 2009; Faust and Logan, 2018). Numerous studies have proven that such SL can noticeably improve both the yield and quality of various high-value specialty crops (Morrow, 2008; Singh et al., 2015). However, it is important to use the correct spectral qualities for each application to maximize plant performance, as many light responses are species-specific (Taulavuori et al., 2016a; Kyriacou et al., 2019; Santin et al., 2021). Despite being costly to purchase, maintain, and operate, commercial greenhouses lighting systems can be used to create the best possible conditions for growth. They can also be used to enhance the natural solar spectrum and impart desirable metabolic effects, such as the accumulation of aroma compounds and phytonutrients with known human health benefits (Rao and Rao, 2007; Poiroux-Gonord et al., 2010; Petrovic et al., 2019). By analyzing the impact of specific wavelengths created by existing greenhouse lighting systems on plant metabolism, we can develop energy-efficient SL strategies to enhance yields and the overall sensory quality of many high-value specialty crops.

One of the most popular and highly valued annual culinary herbs is sweet basil. It has a complex and unique aroma profile desired by professional chefs and restaurants worldwide (Putievsky and Galambosi, 1999; Hiltunen and Holm, 2003). It has a high harvest index and profit margin, is relatively easy to grow, and is well adapted for commercial greenhouse hydroponics and other controlled environment agriculture (CEA) systems (Sipos et al., 2021). The use of greenhouse hydroponics to cultivate basil can provide ideal climate and nutrient conditions that could help diminish any changes in plant growth or development caused by seasonal variations in environmental conditions (Kopsell et al., 2005; Kiferle et al., 2013). Basil is rich in phenolic and terpenoid compounds, many of which are important for human sensory perception and possess human health benefits (Pattison et al., 2018). The ‘Italian Large Leaf’ variety is known for its strong and intense flavor, vigorous growth, and large leaves, with extensive use in Western and Mediterranean cuisines (Shanmugam et al., 2018). Because of its popularity, demand, and intense VOC profile, sweet basil makes for an excellent model crop to explore the interactions of SL, the natural solar spectrum, and secondary metabolic resource allocation.

The intricacy of sweet basil’s taxonomy is due to its hybridization, mislabeling, and abundance of cultivars (Sipos et al., 2021). Recent studies have highlighted that even though these cultivars may look alike and often share the same name, they are genetically distinct from one another. Variations in the genetic background will profoundly impact light-mediated responses associated with secondary metabolism and aroma volatiles (De Masi et al., 2006; Bernhardt et al., 2015). The optimization of basil production in controlled environments depends on many factors, specifically the intensity and spectral quality of light provided.

Light-emitting diodes (LEDs) allow growers to precisely provide discrete narrowband wavelengths to their crops compared to traditional broadband lighting systems (i.e., high-pressure sodium). Spectral manipulation using LEDs can be used to alter the traits of basil, including its biomass and morphology, as well as its biochemical composition during growth and post-harvest (Hasan et al., 2017; Sipos et al., 2020). The potential for this physiological manipulation has been demonstrated with increases in total phenolic and isoprenoid concentrations when using narrowband blue and red light supplements. Research has shown that the addition of yellow and/or green wavelengths to blue and red wavelengths increased several monoterpenes, sesquiterpenes, and phenylpropanoids in basil compared with blue and red wavelength spectra (Stagnari et al., 2018; Sipos et al., 2020; Kivimaenpa et al., 2022). Further, many studies have demonstrated the species-specific (in some cases, even variety-specific) nature of secondary metabolic responses to narrowband wavelengths, warranting further investigation of both phenolics and terpenoids in basil and other high-value specialty crops (Kyriacou et al., 2019; Toscano et al., 2021). Because of their importance in human sensory perception, the phenolic and terpenoid pathways should be thoroughly evaluated using various analytical and molecular techniques, since light-mediated secondary metabolic resource allocation will impact the expression/bioaccumulation of certain compounds as well as entire secondary metabolic pathways. To date, no published scientific investigations have explored the impact of discrete supplemental narrowband wavelengths and broadband lighting sources on the aroma volatile profile of ‘Italian Large Leaf’ basil across the changing natural solar spectrum under glass greenhouses across growing seasons.

With this in mind, we designed a set of experiments to determine the overall impact of spectral quality variation of SL on a common variety of greenhouse-produced hydroponically grown basil. The goals of this project were to (1) explore, identify, and quantify plant volatile organic compounds with known impacts on sensory perception or plant physiological processes of basil using headspace gas chromatography-mass spectrometry (HS GC-MS); (2) determine the impact of spectral quality from ambient sunlight and SL sources on aroma volatile concentrations, including specific ratios of narrowband blue/red wavelengths, combinations of discrete narrowband wavelengths, and broadband wavelength; and (3) provide physiology-based recommendations for lighting regimes (spectral quality of supplemental horticultural lighting systems) for commercial greenhouse basil production.

We hypothesize that discrete waveband supplements will differentially influence specific aroma volatiles and secondary metabolic resource allocation (i.e., particular compounds and classes of compounds). We predict this experiment will confirm that manipulating the spectral quality of SL has a considerable impact on basil volatiles and can potentially enhance the human olfactory experience.

## Materials and methods

### Cultural techniques and environmental growing conditions

This project was conducted at The University of Tennessee Institute of Agriculture (UTIA) in Knoxville, TN, USA (35°56’44.5”N, 83°56’17.3”W). Growing dates for these four experimental runs occurred from January 2019 to October 2019 and have been labeled as growing seasons. *Ocimum Basilicum* var. Italian Large Leaf basil seeds (Johnny’s Select Seeds, Winslow, ME, United States) were germinated in peat moss-based cubes (2 × 2 × 6 cm) (Park’s Bio Dome Sponges, Hodges, SC, United States) at 28.3°C and 95% RH. The ‘Italian Large Leaf’ variety of sweet basil was specifically chosen because of its unique flavor profile, high market demand, high yields, and preference among professional chefs. After two weeks, seedlings were transferred to nutrient film technique (NFT) hydroponic systems with full-strength general mix nutrient solution; the fertility regime was kept constant across the duration of all seasons. The nutrient solution was kept consistent at 5.9 pH and changed weekly. Elemental nutrient concentrations were as follows (ppm): Nitrogen (207.54), Phosphorous (50.87), Potassium (298.23), Calcium (180.15), Magnesium (77.10), Sulfur (136.45), Iron (3.95), Manganese (0.90), Zinc (0.40), Molybdenum (0.09), Copper (0.90), and Boron (0.90). Water samples were analyzed using Inductively Coupled Plasma Mass Spectrometry (Agilent Technologies, Santa Clara, CA, United States) throughout each experiment to ensure consistent nutrient composition. Total growth time lasted approximately 45 d across all four experimental runs (growing seasons). Relative humidity during the growth period averaged 52.5%. Day temperatures averaged 28.5°C, and night temperatures averaged 21.2°C. The DLI of the natural light control averaged 11.75 mol^**.**^m^-2.^d^-1^ during the growth period (ranging from 4 to 20 mol^**.**^m^-2.^d^-1^). Specific growing parameters for each of the seasons may be found in **Table 1**.

**Table 1:** Important environmental parameters across growing cycles.

Growing Period	“January”	“April”	“June”	“September”
	1/7/19-2/18/19	3/25/19-5/08/19	5/15/19-7/02/19	9/3/19-10/14/19
Average Day Temp (°C)	27.8	28.1	29.4	28.9
Average Night Temp (°C)	20.2	21.7	22.2	21.3
Average Relative Humidity	55%	50%	50%	55%
Average Daily Light Integral (DLI) (mol^**.**^m^.-2.^d^-1^)	7.81	10.29	15.65	13.87
Average Day Length (h)	10:02	13:08	14:29	12:11

All crops grown under greenhouse conditions at The University of Tennessee Institute of Agriculture (UTIA) in Knoxville, TN, USA (35°56’44.5”N, 83°56’17.3”W).

**Figure 1:**
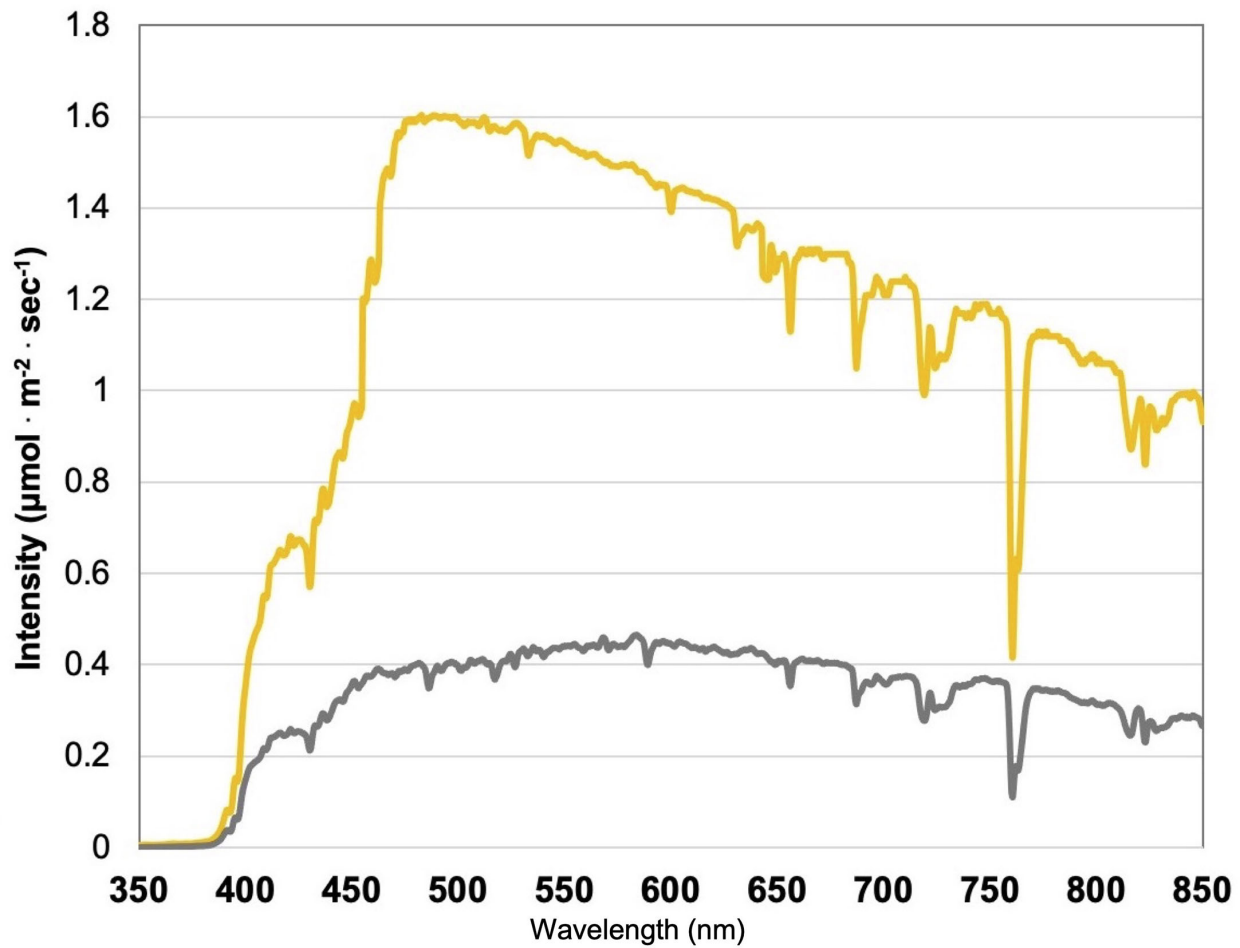
Natural light (NL) spectra under greenhouse glass, averaged across all four growing seasons, ranging from 350 nm to 850 nm. Values were taken at solar noon with three replicates for full sun (yellow) and overcast (gray) for each experimental run. The daily light integral (DLI) of the NL control averaged 11.75 mol^**.**^m^-2.^d^-1^ across all growing cycles (ranging from 4 to 20 mol^**.**^m^-2.^d^-1^).

**Figure 2:**
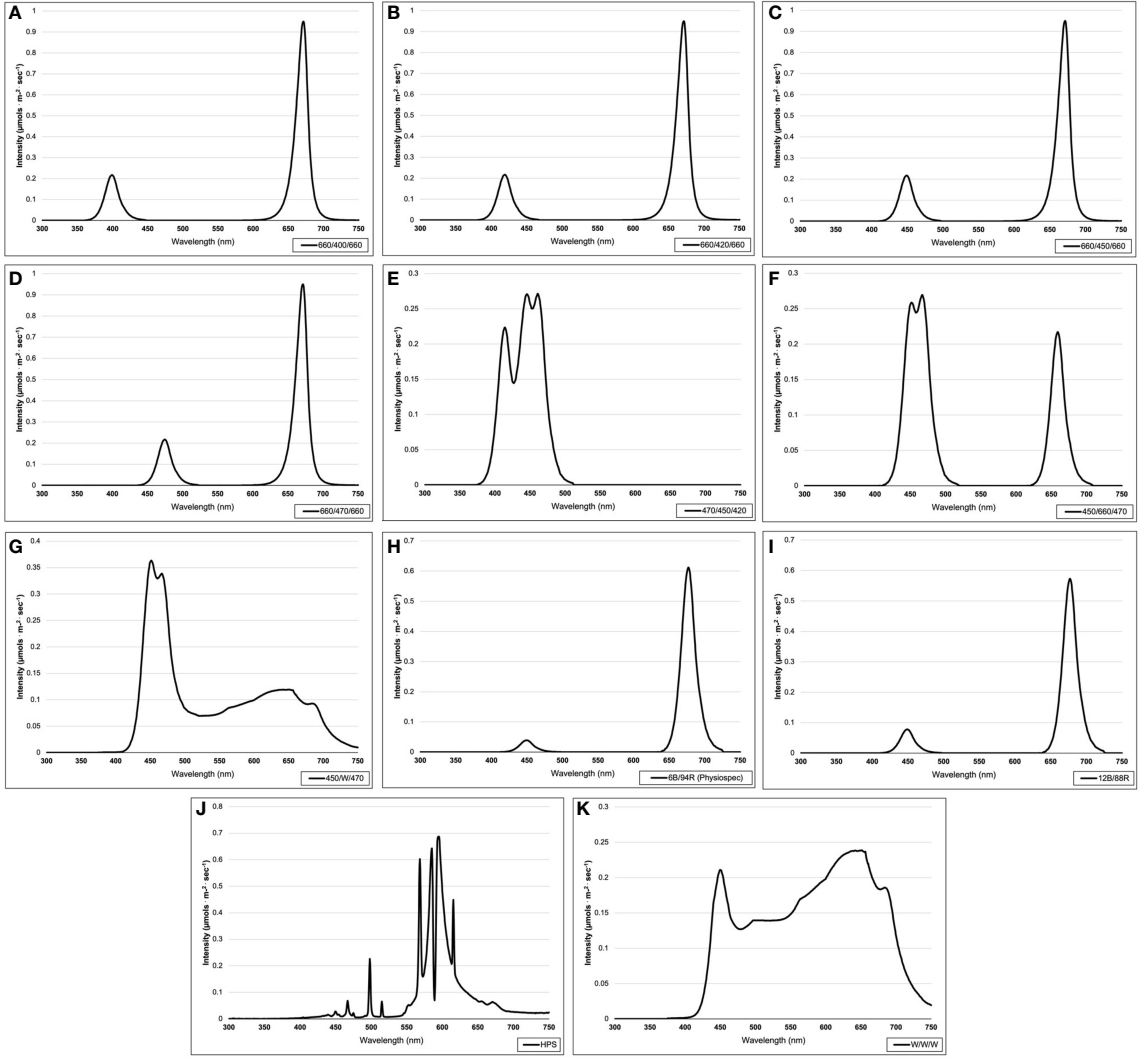
Emission spectra of supplemental lighting (SL) treatments from 300 nm to 750 nm: **(A)** 660/400/660; **(B)** 660/420/660; **(C)** 660/450/660; **(D)** 660/470/ 660; **(E)** 470/450/420; **(F)** 450/660/470; **(G)** 450/W/470; **(H)** 6B/94R; **(I)** 12B/88R; **(J)** HPS; **(K)** W/W/W. All SL treatments provided 8.64 mol·m^-2^·d^-1^ (continuous 100 µmol·m^-2^·s^-1^; 24 h·d^-1^). All lighting treatments were measured with a PS-200 Apogee Spectroradiometer to confirm the intensity of specific treatment wavelengths throughout each growing season. Readings were taken at midnight in order to exclude underlying natural solar spectra.

This experiment evaluated the impact of discrete narrowband wavelength combinations from SL systems on tissue concentrations of plant volatile organic compounds (PVOCs) pertinent to flavor/aroma profile and human sensory perception. A total of 12 lighting treatments were used in this experiment, which included one non-supplemented natural light (NL) control (**Figure 1**) and eleven supplemental lighting (SL) treatments of equal intensity with varying spectral distributions (**Figures 2A–K**). LEDs (Fluence Bioengineering, Austin, TX) and HPS lamps (Hortilux DE, Mentor OH) provided 8.64 mol^**.**^m^-2.^d^-1^ (equal intensity of 100 µmol^**.**^m^-2.^s^-1^ for 24 h^**.**^d^-1^) for each SL treatment, in addition to natural sunlight (**Figure 1**). Lighting treatments are denoted by their wavelengths applied, and each wavelength in series was applied at equal intensities (i.e., a ratio of 1:1:1, with target intensities of 33.3/33.3/33.3 µmol^**.**^m^-2.^s^-1^). The intensity and duration of the lighting treatments in this experiment were selected based on current literature with the intention of maximizing the production of key secondary metabolites known to influence flavor perception in basil.

Four treatments applied narrowband red wavelengths across varying narrowband blue wavelengths (ratio of 1B:2R as 660/400/660, 660/420/660, 660/450/660, and 660/470/660) (Fluence Bioengineering, Austin, TX). One treatment applied a high dose of only narrowband blue wavelengths (470/450/420) (Fluence Bioengineering, Austin, TX), while another applied a moderated amount of narrowband blue wavelengths with some narrowband red wavelengths (ratio of 2B:1R as 450/660/470) (Fluence Bioengineering, Austin, TX). Two PhysioSpec lighting systems (Fluence Bioengineering, Austin, TX) were used to evaluate the ratio of narrowband blue and red wavelengths (ratios of 3B:47R and 3B:22R, as 6B/94R and 12B/88R, respectively). Finally, three broadband supplemental treatments of various color temperatures were used, which included a high blue (450/W/470) (Fluence Bioengineering, Austin, TX), a neutral white (W/W/W) (Fluence Bioengineering, Austin, TX), and a high orange/red (HPS) (Hortilux DE, Mentor OH). As previously stated, all SL treatments were provided at equal intensity and duration. Treatments were measured with an Apogee PS-200 spectroradiometer (Apogee Instruments, Logan UT) multiple times per week (after dark) and regularly adjusted to ensure consistent SL intensities and spectral distributions across growing seasons.

Each SL treatment was physically separated to ensure no bleed-over effects between treatments (average of 1.1 ± 0.6 µmol^**.**^m^-2.^s^-1^ SL bleed-over at the treatment edges). 1.2 m x 1.2 m sections of basil were grown, with 1.2 m separation between treatments (i.e., measurement edge-to-edge of hydroponic systems within the greenhouse). Tissue samples were only harvested from within the middle 0.6 m of each treatment to ensure further reduction of SL contamination between treatments (0.3 m around the edge of each treatment was considered the buffer zone and was not used for sampling). SL bleed-over was <0.1 µmol^**.**^m^-2.^s^-1^ within the harvest zone of each treatment (i.e., below the instrumentation detection limit). Harvests occurred directly after sunrise, and samples were immediately sealed and frozen in liquid nitrogen, then transferred to a -80°C freezer until the time of analysis to preserve all volatile compounds and inhibit post-harvest changes to metabolism.

### Gas chromatography and mass spectrometry method

Three g of fresh leaf tissue (two basil plants per sample rep, 1.5 g of representative material from each plant, nodes four and eight) were placed in 20 mL borosilicate glass vials, then immediately frozen in liquid nitrogen, and stored in a -80 °C freezer until time of analysis. Samples were run within 72 hours of collection. Frozen samples were placed onto a Network Headspace Sampler (Agilent G1888, Santa Clara, CA, United States). Ten sample reps were used per treatment. Samples were heated to 80 °C for 10 min and pressurized with Helium (Air Gas, analytical purity) to 95.21 kPa for 1 min. The tube was then vented for 1 min into the headspace transfer line (110 °C) and injected (port at 250 °C) into the GC (Agilent Technologies 6890N Network GC System). The volatiles were separated by an HP-5MS capillary column ((5%-Phenyl)-methylpolysiloxane, length: 30 m, ID: 0.250 mm, film thickness: 1 µm, Agilent Technologies) using analytical purity Helium carrier gas at 95.21 kPa with constant column pressure. At the start of data acquisition, the temperature was held at 40 °C for 5 min, ramped up from 40 °C to 250 °C (5 °C per min), then held constant for the duration of the run. The total run time was 70 min, including post-run and cool-down phases. After sample separation and column elution, the analytes were passed through a mass selective detector (Agilent Technologies 5973 Network Mass Selective Detector) at 250 °C and collected over the course of the sample run. The transfer line, ion source, and quadrupole temperatures were 250 °C, 230 °C, and 170 °C, respectively. The full scan mass range was set to 40-550 m/z (threshold: 150).

Agilent ChemStation was used for data collection and processing. Over 200 separate compounds were identified throughout this experiment, but emphasis was placed on key aroma compounds (i.e., shown in the literature to be essential for human sensory perception and/or plant metabolic processes) that have been calibrated to our GC-MS and HP-5MS column using pure analytical standards (Sigma-Aldrich, St. Louis, MO) to determine leaf tissue emissions of key VOCs on a fresh plant weight basis. The MS spectra from pure analytical standards and fresh samples were compared to NIST, ADMIS, and our custom basil reference library created from calibrated analytical standards to confirm peak identity and retention times. MassHunter Workstation Software Version B.06.00 (Agilent Technologies, Inc., 2012) was used to integrate peaks automatically. Relative peak areas and retention times were automatically adjusted based on authentic analytical standards and multiple library references. Over 200 compounds were identified in this experiment, with approximately 50 of those being quantified using pure analytical standards.

**Figure 3:**
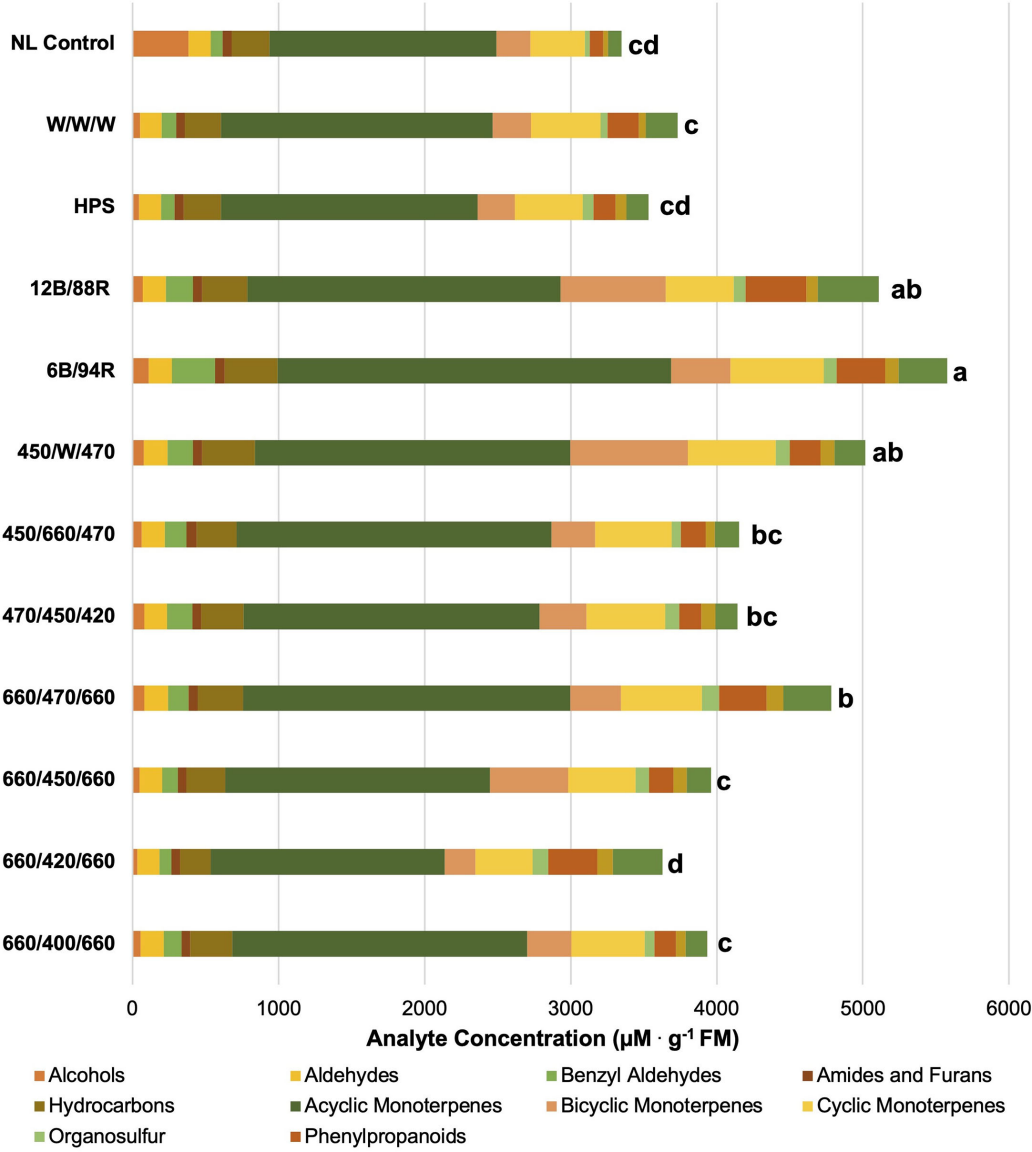
Impact of lighting treatment on tissue accumulation of each compound class. All concentrations are presented in micro molarity of analyte per gram of fresh mass (µM·g^-1^ FM). Mean values represent two plants per replication and ten replications per treatment. Values for each treatment are averaged across all seasons within that treatment. Total volatile organic compound concentration was analyzed using Tukey’s protected least significant difference. Data followed by the same letter are not significantly different (α = 0.05).

**Figure 4:**
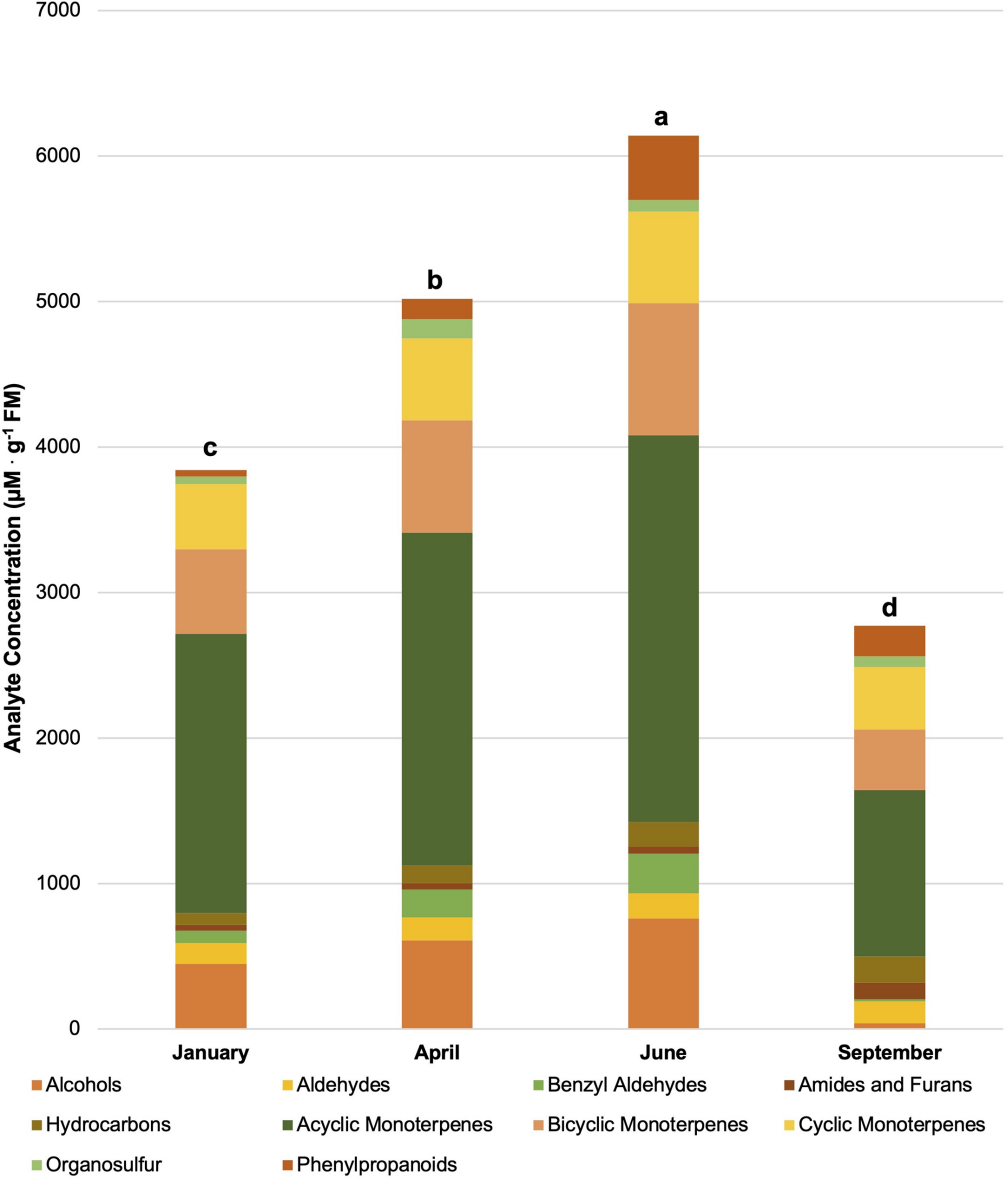
Influence of season on compound classes pertinent for aroma perception. All concentrations are presented in micro molarity of analyte per gram of fresh mass (µM·g^-1^ FM). Mean values represent two plants per replication and ten replications per treatment. Values for each season are averaged across all treatments within that season. Total volatile organic compound concentration was analyzed using Tukey’s protected least significant difference. Data followed by the same letter are not significantly different (α = 0.05).

All volatile concentration units are reported in micro molarity of analyte concentration (suspended in a known volume of gaseous headspace matrix) per g of fresh leaf tissue (µM·g^-1^ FM) to represent VOC emissions most accurately from the collected headspace sample above fresh plant tissues under specific reproducible analytical conditions (**Tables 2**–**4**; **Figures 3**, **4**). This unit (compared to µmol·g^-1^ FM) was utilized because of its commonality in biological headspace GC-MS sampling and incorporates the concentration of each analyte per unit volume of headspace gas above the plant tissue (i.e., samples the dynamic and complex gaseous matrix which contains numerous pertinent VOCs), which is important for sensory-based studies. This provides the foundation for future sensory panel experiments aimed at determining the influence of light on consumer acceptance and preference of basil aroma profiles.

**Table 2:** Summary of statistical results for pertinent aroma volatile compounds detected using headspace gas chromatography-mass spectrometry.

Compound Name	CAS Number		F Value			Pr > F	
		Experiment	Treatment	Experiment*Treatment	Experiment	Treatment	Experiment*Treatment
Alcohols							
(E)-3-Hexen-1-ol	928-96-1	18.44	0.81	0.67	<0.0001	0.6264	0.8682
1-Octen-3-ol	3391-86-4	23.16	3.47	2.32	<0.0001	0.0001	<0.0001
2-Octyn-1-ol	20739-58-6	36.42	10.78	3.58	<0.0001	<0.0001	<0.0001
2-Phenylethanol	60-12-8	44.24	11.18	3.90	<0.0001	<0.0001	<0.0001
Aldehydes							
2-Ethyl-2-butenal	19780-25-7	44.87	1.60	2.26	<0.0001	0.0977	0.0002
2-Hexenal	6728-26-3	47.52	2.94	2.11	<0.0001	0.0009	0.0005
Nonanal	124-19-6	199.07	5.41	2.83	<0.0001	<0.0001	<0.0001
Hexanal	66-25-1	7.82	0.98	0.91	<0.0001	0.4703	0.6094
Benzyl Aldehydes							
3-Ethylbenzaldehyde	34246-54-3	140.11	9.66	3.95	<0.0001	<0.0001	<0.0001
Benzeneacetaldehyde	122-78-1	196.22	19.32	7.76	<0.0001	<0.0001	<0.0001
Amides and Furans							
Benzamide	55-21-0	345.39	2.49	2.14	<0.0001	0.0049	0.0003
N-phenyl-Formamide	103-70-8	143.66	1.95	2.31	<0.0001	0.0323	<0.0001
2-Ethyl-furan	3208-16-0	16.32	1.48	1.83	<0.0001	0.1380	0.0045
2-Pentyl-furan	3777-69-3	4.82	2.63	0.90	0.0026	0.0029	0.6374
Hydrocarbons							
1,4-Cyclohexadiene	628-41-1	80.31	8.46	2.74	<0.0001	<0.0001	<0.0001
2-Cyclopropyl-2-pentene	5457-40-9	58.30	9.00	1.98	<0.0001	<0.0001	0.0013
Decane	124-18-5	158.69	5.79	2.66	<0.0001	<0.0001	<0.0001
(E)-3-Methyl-1,3,5-hexatriene	24587-26-6	6.53	1.78	1.04	0.0003	0.0558	0.4103
1,3-cis,5-cis-Octatriene	40087-62-5	42.07	8.62	1.71	<0.0001	<0.0001	0.0100
1,4-Octadiene	5675-25-2	27.54	3.60	2.18	<0.0001	<0.0001	0.0003
2-Methyl-2-hepten-4-yne	58275-91-5	31.70	9.23	2.07	<0.0001	<0.0001	0.0006
1-Methylcyclohexene	591-49-1	135.93	1.92	1.04	<0.0001	0.0356	0.4174
Cycloheptene	628-92-2	3.18	0.67	1.05	0.0258	0.7661	0.4110
Acyclic Monoterpenes							
2,6-Dimethyl-2,4,6-octatriene	673-84-7	65.54	12.59	3.43	<0.0001	<0.0001	<0.0001
cis-β-Ocimene	3338-55-4	48.61	3.18	1.81	<0.0001	0.0004	0.0048
Citronellyl Acetate	150-84-5	13.31	2.70	1.75	<0.0001	0.0024	0.0085
Linalool	78-70-6	47.53	2.17	1.57	<0.0001	0.0152	0.0262
trans-β-Ocimene	3779-61-1	80.44	6.97	3.19	<0.0001	<0.0001	<0.0001
α-Ocimene	6874-44-8	195.03	1.29	2.00	<0.0001	0.2295	0.0011
β-Myrcene	123-35-3	268.90	5.15	2.83	<0.0001	<0.0001	<0.0001
Bicyclic Monoterpenes							
3-Caren-10-al	14595-13-2	17.21	2.24	1.06	<0.0001	0.0123	0.3871
(+)-4-Carene	29050-33-7	27.75	7.00	1.57	<0.0001	<0.0001	0.0254
3-Carene	498-15-7	24.28	4.53	2.35	<0.0001	0.0521	0.0005
Camphene	79-92-5	70.69	9.41	1.52	<0.0001	<0.0001	0.0356
Isoborneol	507-70-0	22.21	3.85	1.51	<0.0001	<0.0001	0.0367
trans-Pinocarveol	5947-36-4	7.36	3.32	1.89	<0.0001	0.0002	0.00027
trans-Sabinene hydrate	17699-16-0	158.38	5.80	2.66	<0.0001	<0.0001	<0.0001
α-Pinene	80-56-8	73.74	9.40	1.65	<0.0001	<0.0001	0.0152
β-Pinene	127-91-3	49.46	5.93	1.86	<0.0001	<0.0001	0.0032
Cyclic Monoterpenes							
3-Menthene	500-00-5	10.23	4.23	2.34	<0.0001	<0.0001	<0.0001
d-Limonene	5989-27-5	29.80	7.17	1.74	<0.0001	<0.0001	0.0076
Fenchyl acetate	13851-11-1	14.98	1.58	0.62	<0.0001	0.1019	0.9467
p-Menth-1-en-8-ol	98-55-5	173.75	10.61	2.56	<0.0001	<0.0001	<0.0001
Sesquiterpenes							
a-Humulene	6753-98-6	19.58	1.87	1.12	<0.0001	0.0420	0.3001
Organosulfur							
Isothiocyanatocyclopropane	56601-42-4	9.92	1.69	0.85	<0.0001	0.0752	0.7103
2-Isobutylthiazole	18640-74-9	10.79	1.90	1.23	<0.0001	0.0391	0.2101
Diallyl Disulfide	2179-57-9	1.69	0.40	0.67	0.1684	0.9559	0.9145
Dimethyl Sulfide	75-18-3	25.15	4.30	2.09	<0.0001	<0.0001	0.0005
Phenylpropanoids							
Eugenol	97-53-0	130.10	9.94	4.43	<0.0001	<0.0001	<0.0001
Methyl Eugenol	93-15-2	7.95	1.70	1.50	0.0010	0.0997	0.1545

**Table 3:** Influence of growing season on aroma volatile tissue concentrations in hydroponically grown greenhouse sweet basil (*Ocimum basilicum* var. Italian Large Leaf).

Compound Name	Growing Season			
	January	April	June	September
Alcohols				
(E)-3-Hexen-1-ol	2.27^b^	2.07^b^	6.24^a^	5.99^a^
1-Octen-3-ol	16.43^bc^	23.23^b^	39.72^a^	13.03^c^
2-Octyn-1-ol	408.20^c^	547.80^b^	653.76^a^	395.22^d^
2-Phenylethanol	19.56^c^	35.55^b^	58.43^a^	26.41^bc^
Aldehydes				
2-Ethyl-2-butenal	0.87^c^	1.11^bc^	1.37^bc^	2.32^a^
2-Hexenal	3.39^c^	4.43^bc^	5.39^b^	9.69^a^
Nonanal	10.05^c^	20.82^b^	37.32^a^	5.26^d^
Hexanal	128.96^c^	130.21^bc^	131.06^ab^	132.15^a^
Benzyl Aldehydes				
3-Ethylbenzaldehyde	5.82^c^	10.14^b^	17.15^a^	4.28^c^
Benzeneacetaldehyde	76.12^c^	181.19^b^	250.85^a^	9.92^d^
Amides and Furans				
Benzamide	2.92^b^	3.30^b^	3.49^b^	36.32^a^
N-phenyl-Formamide	6.92^b^	6.32^b^	9.05^b^	39.48^a^
2-Ethyl-furan	27.51^c^	29.83^b^	29.59^b^	32.22^a^
2-Pentyl-furan	4.83^b^	5.97^ab^	6.33^a^	5.27^ab^
Hydrocarbons				
1,4-Cyclohexadiene	6.00^c^	10.31^b^	17.34^a^	16.76^a^
2-Cyclopropyl-2-pentene	12.09^c^	20.43^b^	24.59^a^	25.58^a^
Decane	9.99^c^	20.71^b^	37.07^a^	10.61^c^
(E)-3-Methyl-1,3,5-hexatriene	2.00^b^	2.15^b^	2.67^ab^	3.19^a^
1,3-cis,5-cis-Octatriene	5.41^b^	6.27^b^	8.84^a^	8.20^a^
1,4-Octadiene	16.63^bc^	23.04^b^	40.63^a^	7.28^c^
2-Methyl-2-hepten-4-yne	6.56^b^	6.80^b^	8.85^a^	9.08^a^
1-Methylcyclohexene	23.40^b^	29.14^b^	32.83^b^	89.07^a^
Cycloheptene	0.73^b^	0.81^b^	1.02^a^	1.09^a^
Acyclic monoterpenes				
2,6-Dimethyl-2,4,6-octatriene	1.77^d^	4.16^c^	6.30^a^	5.15^b^
cis-β-Ocimene	370.45^c^	538.33^b^	754.18^a^	145.81^d^
Citronellyl Acetate	188.27^c^	190.24^bc^	193.66^a^	191.75^b^
Linalool	925.12^a^	876.30^a^	870.25^a^	239.32^b^
trans-β-Ocimene	106.90^c^	279.90^b^	351.14^a^	57.16^c^
α-Ocimene	45.66^b^	27.88^b^	75.23^b^	493.99^a^
β-Myrcene	281.89^b^	371.46^a^	407.65^a^	9.36^c^
Bicyclic monoterpenes				
3-Caren-10-al	1.79^b^	1.21^c^	1.52^bc^	2.51^a^
(+)-4-Carene	16.93^d^	26.22^c^	61.46^a^	40.74^b^
3-Carene	379.21^b^	507.44^a^	559.24^a^	535.31^a^
Camphene	23.41^c^	29.25^bc^	32.83^bc^	55.77^a^
Isoborneol	28.97^bc^	23.53^c^	32.50^b^	49.74^a^
trans-Pinocarveol	3.60^b^	4.89^ab^	5.87^a^	6.13^a^
trans-Sabinene hydrate	10.04^c^	20.71^b^	37.07^a^	10.60^c^
α-Pinene	17.27^c^	22.81^b^	25.94^b^	46.27^a^
β-Pinene	99.30^c^	137.69^b^	152.76^b^	208.12^a^
Cyclic monoterpenes				
3-Menthene	24.22^b^	36.08^a^	46.11^a^	39.59^a^
d-Limonene	234.61^c^	311.32^b^	378.51^a^	315.24^b^
Fenchyl acetate	132.80^a^	149.80^a^	127.37^a^	81.12^b^
p-Menth-1-en-8-ol	5.44^d^	9.75^c^	19.69^a^	13.33^b^
Sesquiterpenes				
α-Humulene	53.69^b^	54.88^b^	58.92^a^	59.58^a^
Organosulfur				
Isothiocyanatocyclopropane	1.28^b^	1.20^b^	1.78^ab^	2.28^ab^
2-Isobutylthiazole	5.57^b^	6.74^b^	8.86^a^	8.94^a^
Diallyl Disulfide	0.65^a^	0.87^a^	1.05^a^	1.60^a^
Dimethyl Sulfide	43.42^c^	121.98^a^	68.87^b^	69.76^b^
Phenylpropanoids				
Eugenol	43.16^d^	139.70^c^	280.02^a^	212.33^b^
Methyl Eugenol	131.23^b^	138.51^b^	160.17^a^	172.11^a^

All concentrations are presented in micro molarity of analyte per gram of fresh mass (µM·g^-1^ FM). Mean values represent two plants per replication and ten replications per treatment. Values for each season are averaged across all treatments within that season. Values were analyzed using Tukey’s protected least significant difference. Data in the same row followed by the same letter are not significantly different (α = 0.05).

### Statistical analyses

A Randomized Complete Block Design was used for this experiment. All data sets were analyzed by Generalized Linear Model (GLM) and Mixed Model Analysis of Variance (p = 0.05) procedures using the statistical software SAS (version 9.4, SAS Institute, Cary, NC). Design and Analysis macro (DandA.sas), created by Dr. Arnold Saxton, was utilized in addition to Tukey’s adjustment, regression analysis, and univariate/normalization procedures to provide additional statistical insights on the complete data set. Treatments were separated by least significant difference (LSD) at α=0.05. Principal component analysis was performed using JMP Pro 17 (SAS Institute, Cary, NC). Due to the overwhelming number of compounds analyzed, only statically significant separations of compounds with known plant physiological function and/or human sensory impact were reported in this study. Key volatiles were analyzed and presented on a fresh mass (FM) basis as compared to calibration curves created from pure analytical standards.

## Results

Plant volatile organic compound (PVOC) leaf tissue concentrations were evaluated in this experiment, many of which were influenced by growing season, lighting treatment, and season*treatment interactions. Total VOC concentrations of basil leaf tissues were found to be statistically significant across both lighting treatment (F=103.01; *P ≤* 0.0001) (**Figure 3**) and season (F=391.62; *P ≤* 0.0001) (**Figure 4**). Statistical summary for individual compounds evaluated in this study are included (**Table 1**) and separated by category based on chemical class and metabolic origin. Categories include alcohols, aldehydes, benzyl aldehydes, amides and furans, hydrocarbons, acyclic monoterpenes, bicyclic monoterpenes, cyclic monoterpenes, sesquiterpenes, organosulfur, and phenylpropanoids.

### Alcohols

(E)-3-Hexen-1-ol concentrations were significantly impacted by season (F=18.44; *P ≤* 0.0001), but not by lighting treatment (F=0.81; *P=*0.6264) or season*treatment interactions (F=0.67; *P=*0.8682) (**Table 2**). When averaged across all treatments, June and September had higher average concentrations, as compared to the January and April seasons. Season concentrations ranged from 2.27 µM·g^-1^ FM to 6.24 µM·g^-1^ FM (**Table 3**).

1-Octen-3-ol concentrations were significantly influenced by season (F=23.16; *P ≤* 0.0001), lighting treatment (F=3.47; *P ≤* 0.0001), and season*treatment interactions (F=2.32; *P ≤* 0.0001) (**Table 2**). The June growing season had the highest concentration averages as compared to any other season. September had the lowest but did not separate from January. Season concentrations ranged from 13.03 µM·g^-1^ FM to 39.72 µM·g^-1^ FM (**Table 3**). When averaged across all seasons, treatments 450/W/470 and 660/470/660 statistically separated from the 660/420/660 treatment, but the others did not show clear separation. Treatment concentration averages ranged from 15.15 µM·g^-1^ FM to 35.65 µM·g^-1^ FM (**Table 4**).

**Table 4:** Influence of light treatment on aroma volatile tissue concentrations in hydroponically grown greenhouse sweet basil (*Ocimum basilicum* var. Italian Large Leaf).

Compound Name	Lighting Treatments											
	660/400/660	660/420/660	660/450/660	660/470/660	470/450/420	450/660/470	450/W/470	6B/94R	12B/88R	HPS	W/W/W	NL Control
Alcohols												
(E)-3-Hexen-1-ol	4.45^a^	2.90^a^	5.78^a^	3.32^a^	28.15^a^	4.57^a^	4.06^a^	3.66^a^	1.97^a^	4.33^a^	2.88^a^	2.54^a^
1-Octen-3-ol	20.48^abc^	10.40^c^	15.17^bc^	36.11^a^	16.03^bc^	24.75^abc^	35.65^a^	31.25^ab^	24.65^abc^	18.57^abc^	20.41^abc^	24.70^abc^
2-Phenylethanol	29.64^bcd^	20.70^d^	28.35^bcd^	42.26^bc^	36.71^bcd^	33.43^bcd^	37.98^bcd^	74.13^a^	44.78^b^	19.62^d^	26.80^bcd^	24.47^cd^
Aldehydes												
2-Ethyl-2-butenal	1.51^a^	1.12^a^	1.14^a^	1.36^a^	1.70^a^	1.26^a^	1.42^a^	1.43^a^	1.26^a^	1.39^a^	1.47^a^	2.15^a^
2-Hexenal	5.84^ab^	4.35^b^	4.72^b^	5.98^ab^	4.85^b^	5.26^b^	6.15^ab^	5.82^ab^	5.03^b^	5.55^b^	5.84^ab^	9.00^a^
Nonanal	20.31^abcd^	15.37^cde^	16.10^bcde^	24.48^a^	18.40^abcde^	18.82^abcde^	23.03^abc^	24.14^ab^	18.75^bcde^	14.58^de^	14.18^de^	12.02^e^
Hexanal	131.48^a^	131.12^a^	130.58^a^	130.03^a^	131.23^a^	132.24^a^	131.00^a^	129.98^a^	130.56^a^	131.11^a^	128.63^a^	130.84^a^
Benzyl Aldehydes												
3-Ethylbenzaldehyde	8.94^bcd^	5.35^d^	7.71^cd^	11.48^abc^	8.96^bcd^	8.44^bcd^	10.98^bc^	15.18^a^	11.80^abc^	7.98^bcd^	8.36^bcd^	6.91^d^
Benzeneacetaldehyde	112.53^bcde^	72.21^e^	97.43^cde^	126.75^bcde^	161.13^b^	136.60^bcd^	158.82^bc^	276.22^a^	172.59^b^	83.91^de^	86.51^de^	69.70^e^
Amides and Furans												
Benzamide	9.97^ab^	8.40^b^	9.94^ab^	10.23^ab^	14.69^ab^	12.25^ab^	16.33^a^	14.29^ab^	11.89^ab^	9.73^ab^	11.13^ab^	9.49^ab^
N-phenyl-Formamide	12.85^ab^	16.24^ab^	15.69^ab^	16.85^ab^	9.87^b^	22.42^a^	11.66^ab^	15.04^ab^	15.37^ab^	15.91^ab^	14.69^ab^	18.73^ab^
2-Ethyl-furan	29.05^a^	29.77^a^	28.40^a^	28.63^a^	31.32^a^	31.16^a^	30.66^a^	29.05^a^	28.99^a^	29.68^a^	30.07^a^	30.75^a^
2-Pentyl-furan	5.74^ab^	4.80^ab^	5.02^ab^	6.52^ab^	5.38^ab^	5.02^ab^	6.79^ab^	7.04^a^	6.19^ab^	5.51^ab^	4.81^ab^	4.54^b^
Hydrocarbons												
1,4-Cyclohexadiene	12.21^bcd^	6.38^d^	10.39^cd^	14.17^abc^	12.84^bcd^	11.24^bcd^	16.01^ab^	18.66^a^	15.92^ab^	10.67^cd^	11.74^bcd^	9.16^d^
2-Cyclopropyl-2-pentene	19.42^cdef^	14.51^f^	17.05^def^	24.41^abc^	23.26^abcd^	20.81^bcdef^	26.17^ab^	27.61^a^	22.97^abcde^	17.43^def^	17.71^def^	16.74^ef^
Decane	21.66^abc^	17.05^bcd^	16.91^bcd^	25.92^a^	19.65^abcd^	19.96^abcd^	24.46^ab^	25.57^a^	19.90^abcd^	15.52^cd^	15.58^cd^	13.10^d^
(E)-3-Methyl-1,3,5-hexatriene	2.33^a^	1.78^a^	2.25^a^	2.28^a^	2.50^a^	2.81^a^	2.59^a^	3.37^a^	3.38^a^	2.30^a^	1.95^a^	2.56^a^
1,3-cis,5-cis-Octatriene	6.81^cd^	5.08^d^	5.97^cd^	7.67^bc^	7.42^bc^	6.61^cd^	8.82^ab^	9.77^a^	7.86^abc^	6.49^cd^	7.55^bc^	6.19^cd^
1,4-Octadiene	19.76^abc^	8.98^c^	14.86^bc^	35.66^a^	16.99^abc^	24.25^abc^	35.60^a^	31.24^ab^	22.96^abc^	17.61^abc^	20.19^abc^	14.70^abc^
2-Methyl-2-hepten-4-yne	7.40^cde^	5.52^e^	6.74^de^	8.27^bcd^	7.92^bcd^	7.34^cde^	9.44^ab^	10.20^a^	8.72^abc^	7.30^cde^	7.96^bcd^	7.17^cde^
1-Methylcyclohexene	198.20^a^	153.05^a^	190.83^a^	191.56^a^	200.03^a^	181.60^a^	235.55^a^	234.54^a^	207.13^a^	179.01^a^	163.04^a^	188.81^a^
Acyclic Monoterpenes												
2,6-Dimethyl-2,4,6-octatriene	3.89^bcde^	2.62^e^	3.41^cde^	4.15^bcde^	5.39^b^	4.76^bcd^	5.27^bc^	7.65^a^	5.56^b^	3.20^de^	3.78^bcde^	2.55^e^
cis-β-Ocimene	454.45^b^	345.75^b^	474.37^ab^	435.78^b^	442.53^b^	526.31^ab^	446.03^b^	756.25^a^	489.82^ab^	380.42^b^	331.10^b^	343.47^b^
Citronellyl Acetate	191.03^ab^	189.75^b^	190.81^ab^	189.65^b^	189.24^b^	191.93^ab^	191.62^ab^	194.78^a^	190.54^b^	191.23^ab^	191.00^ab^	190.14^ab^
Linalool	753.22^ab^	602.95^b^	628.48^ab^	924.86^a^	696.32^ab^	714.47^ab^	767.72^ab^	848.92^ab^	779.51^ab^	650.81^ab^	778.87^ab^	586.82^ab^
trans-β-Ocimene	195.86^bcd^	111.37^d^	168.39^bcd^	239.57^abc^	216.57^bcd^	232.42^bcd^	262.02^ab^	357.86^a^	205.60^bcd^	119.00^cd^	158.82^bcd^	117.86^cd^
α-Ocimene	152.20^a^	144.29^a^	122.17^a^	140.02^a^	186.90^a^	212.20^a^	186.24^a^	183.96^a^	173.81^a^	164.77^a^	163.11^a^	98.66^a^
β-Myrcene	268.91^abcd^	203.27^d^	225.35^bcd^	308.12^ab^	287.72^abcd^	271.36^abcd^	301.56^abc^	345.31^a^	301.92^abc^	245.65^bcd^	235.39^bcd^	216.54^cd^
Bicyclic Monoterpenes												
3-Caren-10-al	1.70^ab^	1.54^ab^	1.54^ab^	1.77^ab^	1.61^ab^	1.66^ab^	2.31^a^	2.40^a^	1.58^ab^	1.75^ab^	1.95^ab^	1.32^b^
(+)-4-Carene	24.34^bc^	18.67^c^	22.85^bc^	35.92^ab^	32.04^abc^	31.81^abc^	40.76^a^	45.46^a^	30.60^abc^	21.72^bc^	21.06^bc^	20.90^bc^
Camphene	35.12^bcd^	22.72^d^	30.86^bcd^	41.10^ab^	35.81^bcd^	30.27^bcd^	51.23^a^	51.21^a^	38.04^abc^	28.27^bcd^	32.50^bcd^	26.70^cd^
Isoborneol	32.04^abc^	22.05^c^	29.66^bc^	37.27^abc^	28.57^bc^	33.55^abc^	44.80^ab^	51.35^a^	32.38^abc^	30.87^bc^	37.14^abc^	24.62^c^
trans-Pinocarveol	4.77^ab^	3.12^b^	3.67^b^	4.68^ab^	5.39^ab^	5.68^ab^	6.46^ab^	6.36^ab^	7.30^a^	4.19^ab^	3.66^b^	6.27^ab^
trans-Sabinene hydrate	21.66^abc^	17.05^bcd^	16.98^bcd^	25.92^a^	19.65^abcd^	19.91^abcd^	24.47^ab^	25.63^a^	19.90^abcd^	15.51^cd^	15.57^cd^	13.09^d^
α-Pinene	28.39^bcd^	16.60^d^	23.75^bcd^	32.82^ab^	29.58^bc^	24.52^bcd^	41.75^a^	41.74^a^	30.10^abc^	22.02^bcd^	25.19^bcd^	20.42^cd^
β-Pinene	155.77^abcd^	111.86^d^	117.45^cd^	167.90^abc^	170.62^ab^	153.17^abcd^	190.27^a^	187.17^a^	158.70^abcd^	134.33^bcd^	125.50^bcd^	120.90^bcd^
Cyclic Monoterpenes												
3-Menthene	35.99^abcd^	20.38^d^	25.47^bcd^	31.81^abcd^	41.70^abcd^	39.30^abcd^	45.50^abc^	48.01^ab^	53.10^a^	29.57^bcd^	24.96^cd^	42.28^abcd^
d-Limonene	308.17^bcd^	224.30^d^	271.77^cd^	343.69^abc^	331.01^abc^	301.89^bcd^	371.93^ab^	401.22^a^	341.25^abc^	281.49^cd^	278.99^cd^	263.32^cd^
Fenchyl acetate	92.03^a^	81.94^a^	97.62^a^	111.13^a^	95.53^a^	115.00^a^	109.94^a^	111.16^a^	112.12^a^	86.90^a^	105.84^a^	81.22^a^
p-Menth-1-en-8-ol	10.99^cdef^	7.78^f^	9.53^ef^	13.32^bcd^	12.78^bcde^	11.46^bcdef^	15.01^ab^	17.03^a^	14.39^abc^	10.77^cdef^	11.64^bcde^	10.02^def^
Sesquiterpene												
a-Humulene	55.29^a^	55.99^a^	55.79^a^	56.25^a^	59.41^a^	59.01^a^	57.52^a^	58.82^a^	56.25^a^	56.05^a^	54.51^a^	56.36^a^
Organosulfur												
Isothiocyanatocyclopropane	1.22^a^	1.51^a^	2.03^a^	1.71^a^	1.88^a^	1.67^a^	1.88^a^	2.32^a^	1.66^a^	1.40^a^	1.40^a^	0.99^a^
Diallyl Disulfide	0.87^a^	0.70^a^	0.88^a^	0.95^a^	0.99^a^	1.02^a^	1.21^a^	1.36^a^	0.97^a^	0.80^a^	2.05^a^	0.76^a^
Dimethyl Sulfide	64.89^abc^	104.39^a^	88.73^ab^	112.47^a^	93.37^ab^	59.82^abc^	88.37^ab^	86.28^ab^	70.26^abc^	65.26^abc^	45.70^bc^	32.68^c^
Phenylpropanoids												
Eugenol	146.53^bcd^	178.05^bc^	166.69^bc^	164.96^bc^	151.49^bcd^	116.37^cd^	208.47^b^	283.16^a^	202.84^b^	151.32^bcd^	165.70^bc^	90.09^d^
Methyl Eugenol	158.96^a^	159.54^a^	158.58^a^	165.77^a^	159.72^a^	159.72^a^	160.49^a^	160.12^a^	160.38^a^	162.91^a^	158.11^a^	162.05^a^

All concentrations are presented in micro molarity of analyte per gram of fresh mass (µM·g^-1^ FM). Mean values represent two plants per replication and ten replications per treatment. Values for each treatment are averaged across all four growing seasons. Values were analyzed using Tukey’s protected least significant difference. Data in the same row followed by the same letter are not significantly different (α = 0.05).

2-Octyn-1-ol concentrations were significantly impacted by season (F=36.42; *P ≤* 0.0001), lighting treatment (F=10.78; *P ≤* 0.0001), and season*treatment interactions (F=3.58; *P ≤* 0.0001) (**Table 2**). June again had the highest concentration as compared to any other season. September again had the lowest concentration. Season concentrations ranged from 395.22 µM·g^-1^ FM to 653.76 µM·g^-1^ FM (**Table 3**). While there was statistical separation for the two compounds across lighting treatments, no clear patterns were evident (**Table 4**).

2-Phenylethanol concentrations were significantly influenced by season (F=44.24; *P ≤* 0.0001), lighting treatment, (F=11.18; *P ≤* 0.0001) and season*treatment interactions (F=3.9; *P ≤* 0.0001) (**Table 2**). June again had the highest concentration as compared to any other season. September again had the lowest concentration but did not statistically separate from January. Season concentrations ranged from 19.56 µM·g^-1^ FM to 58.43 µM·g^-1^ FM (**Table 3**). The 6B/94R treatment had the highest concentration and statistically separated from all other treatments. The lowest concentration was found in the 660/420/660 and HPS treatments, but they were not statistically separate from many other treatments. Treatment concentrations ranged from 19.62 µM·g^-1^ FM to 74.13 µM·g^-1^ FM (**Table 4**).

### Benzyl aldehydes

3-Ethylbenzaldehyde concentrations were significantly influenced by season (F=140.11; *P ≤* 0.0001), lighting treatment (F=9.66; *P ≤* 0.0001), and season*treatment interactions (F=3.95; *P ≤* 0.0001) (**Table 2**). The June growing season had the highest concentration and was statistically separated from the lowest seasons, which were January and September. The season concentrations ranged from 4.28 µM·g^-1^ FM to 17.15 µM·g^-1^ FM (**Table 3**). The 6B/94R treatment had the highest concentration and was statistically separated from many other treatments; this includes 660/420/660, 660/450/660, and the NL control, which had three of the lowest concentrations and did not statistically separate. The treatment concentrations ranged from 5.35 µM·g^-1^ FM to 15.18 µM·g^-1^ FM (**Table 4**).

Benzeneacetaldehyde concentrations were significantly influenced by season (F=196.22; *P ≤* 0.0001), lighting treatment (F=19.32; *P ≤* 0.0001), and season*treatment interactions (F=7.76; *P ≤* 0.0001) (**Table 2**). The June growing season had the highest concentration and was statistically separated from the lowest season, which was September. The season concentrations had a considerable range, from 9.92 µM·g^-1^ FM to 250.85 µM·g^-1^ FM (**Table 3**). The 6B/94R again had the highest concentration and statistically separated from all other treatments. In general, the narrowband treatments had higher concentrations than the broadband treatments. NL control had the lowest concentration, but did not separate from the 660/420/660, HPS, and W/W/W treatments. The treatment concentrations ranged from 69.70 µM·g^-1^ FM to 276.22 µM·g^-1^ FM (**Table 4**).

### Hydrocarbons

1,4-Cyclohexadiene concentrations were significantly influenced by growing season (F=80.31; *P ≤* 0.0001), lighting treatment (F=8.46; *P ≤* 0.0001), and season*treatment interactions (F=2.74; *P ≤* 0.0001) (**Table 2**). June and September growing seasons had statistically higher tissue concentrations than those in January and April. The January growing season had the lowest concentrations. The growing season concentrations ranged from 6.00 µM·g^-1^ FM to 17.34 µM·g^-1^ FM (**Table 3**). The treatment 6B/94R again had the highest tissue concentration, statistically greater than many of the narrowband and broadband SL treatments. The 660/420/660 again had the lowest tissue concentration of any treatment, and did not statistically separate from the NL control. The treatment tissue concentrations ranged from 6.38 µM·g^-1^ FM to 18.66 µM·g^-1^ FM (**Table 4**).

2-Cyclopropyl-2-pentene concentrations were significantly influenced by season (F=58.3; *P ≤* 0.0001), treatment (F=9.00; *P ≤* 0.0001), and season*treatment interactions (F=1.98; *P=*0.0013) (**Table 2**). This compound showed similar seasonal patterns to previous hydrocarbons. June and September growing seasons had statistically higher tissue concentrations than those in January and April. The January growing season again had the lowest concentrations. The season concentrations ranged from 12.09 µM·g^-1^ FM to 25.58 µM·g^-1^ FM (**Table 3**). The 6B/94R treatment again had the highest tissue concentration, separating from some of the other treatments. The 660/420/660 had the lowest tissue concentration of any treatment, and did not statistically separate from the NL control and some of the SL treatments. The treatment concentrations ranged from 14.51 µM·g^-1^ FM to 27.61 µM·g^-1^ FM (**Table 4**).

Decane concentrations were significantly influenced by season (F=158.69; *P ≤* 0.0001), treatment (F=5.79; *P ≤* 0.0001), and season*treatment interactions (F=2.66; *P ≤* 0.0001) (**Table 2**). June growing season had the highest tissue concentration, which statistically separated from all other seasons. The two seasons with the lowest concentrations were January and September. The season concentrations ranged from 9.99 µM·g^-1^ FM to 37.07 µM·g^-1^ FM (**Table 4**). The 660/470/660 and 6B/94R treatments had the two highest tissue concentrations, and did not separate from each other. The broadband treatments generally did not have as high concentrations as the narrowband treatments; the HPS and W/W/W treatments did not separate from the NL control, which had the lowest concentration. The treatment concentrations ranged from 13.10 µM·g^-1^ FM to 25.92 µM·g^-1^ FM (**Table 4**).

(E)-3-Methyl-1,3,5-hexatriene concentrations were significantly influenced by season (F=6.53; *P=*0.0003), but not by lighting treatment (F=1.78; *P=*0.0558) or season*treatment interactions (F=1.04; *P=*0.4103) (**Table 2**). The September growing season had the highest tissue concentration, but did not statistically separate from the June season. The January growing season had the lowest tissue concentration but did not separate from the April or June seasons. The season concentrations ranged from 2.00 µM·g^-1^ FM to 3.19 µM·g^-1^ FM (**Table 3**).

1,3-cis,5-cis-Octatriene concentrations were significantly influenced by season (F=42.07; *P ≤* 0.0001), lighting treatment (F=8.62; *P ≤* 0.0001), and season*treatment interactions (F=1.71; *P=*0.01) (**Table 2**). The June growing season had the highest tissue concentration, but did not statistically separate from the September season. The January growing season had the lowest tissue concentration but did not separate from the April season. The season concentrations ranged from 5.41 µM·g^-1^ FM to 8.84 µM·g^-1^ FM (**Table 3**). The 6B/94R treatment had the highest tissue concentration, but did not separate from the 450/W/470 or 12B/88R treatments. The 660/420/660 again had the lowest tissue concentration of any other treatment, and did not statistically separate from the NL control and some of the LED treatments. The treatment concentrations ranged from 5.08 µM·g^-1^ FM to 9.77 µM·g^-1^ FM (**Table 4**).

1,4-Octadiene concentrations were significantly influenced by season (F=27.54; *P ≤* 0.0001), lighting treatment (F=3.60; *P ≤* 0.0001), and season*treatment interactions (F=2.18; *P=*0.0003) (**Table 2**). The June growing season had the highest tissue concentration. The September growing season had the lowest tissue concentration but did not separate from the January season. The season concentrations ranged from 7.28 µM·g^-1^ FM to 40.63 µM·g^-1^ FM (**Table 3**). The treatments 660/470/660 and 450/W/470 had the highest two tissue concentrations and did not statistically from each other, as well many of the other treatments and control. The lowest tissue concentrations were found in the 660/420/660 treatment, which only statistically separated from the highest two treatments in addition to the 6B/94R treatment. The treatment concentrations ranged from 8.98 µM·g^-1^ FM to 35.66 µM·g^-1^ FM (**Table 4**).

2-Methyl-2-hepten-4-yne concentrations were significantly influenced by season (F=31.70; *P ≤* 0.0001), lighting treatment (F=9.23; *P ≤* 0.0001), and season*treatment interactions (F=2.07; *P=*0.0006) (**Table 2**). The September growing season had the highest tissue concentration, but did not statistically separate from the June season. The January growing season had the lowest tissue concentration but did not separate from the April season. The season concentrations ranged from 6.56 µM·g^-1^ FM to 9.08 µM·g^-1^ FM (**Table 3**). The treatment 6B/94R had the highest tissue concentration, but did not separate from the 450/W/470 and 12B/88R treatments. The lowest tissue concentrations were again found in the 660/420/660 treatment, which did not separate from the NL control and some of the LED treatments. The treatment concentrations ranged from 5.52 µM·g^-1^ FM to 10.20 µM·g^-1^ FM (**Table 4**).

1-Methylcyclohexene concentrations were significantly influenced by season (F=135.93; *P ≤* 0.0001) and lighting treatment (F=1.92; *P=*0.0356), but not by season*treatment interactions (F=1.04; *P=*0.4174) (**Table 2**). The September growing season had the highest tissue concentrations, which separated from all other seasons. The lowest tissue concentrations were found during the January growing season, but did not separate from the April and June seasons. The season concentrations ranged from 23.40 µM·g^-1^ FM to 89.07 µM·g^-1^ FM (**Table 3**). The treatment concentrations ranged from 179.01 µM·g^-1^ FM to 235.55 µM·g^-1^ FM; while the p-value from ANOVA did pass the 0.05 threshold, Tukey’s protected LSD test did not indicate separation of means across treatments (**Tables 2**, **4**).

Cycloheptene concentrations were significantly influenced by season (F=3.18; *P=*0.0258), but not by lighting treatment (F=0.67; *P=*0.7661) or season*treatment interactions (F=1.05; *P=*0.411) (**Table 2**). This compound was found in very low concentrations. The June and September growing seasons had the two highest concentrations and did not statistically separate. The January and April growing seasons had the two lowest concentrations and did not statistically separate. The season concentrations ranged from 0.73 µM·g^-1^ FM to 1.09 µM·g^-1^ FM (**Table 3**). Treatment concentrations ranged from 0.70 µM·g^-1^ FM to 1.01 µM·g^-1^ FM (**Table 4**).

### Acyclic monoterpenes

2,6-Dimethyl-2,4,6-octatriene concentrations were significantly influenced by season (F=65.54; *P ≤* 0.0001), lighting treatment (F=12.59; *P ≤* 0.0001), and season*treatment interactions (F=3.43; *P ≤* 0.0001) (**Table 2**). The June growing season had the highest tissue concentration, while the lowest concentration was found in January. Each of the growing seasons statistically separated. The season concentrations ranged from 1.77 µM·g^-1^ FM to 6.30 µM·g^-1^ FM (**Table 3**). The 6B/94R treatment had the highest tissue concentration, which statistically separated from all other treatments. The NL control had the lowest concentration of any treatment, and did not statistically separate from many of the LED treatments as well as the HPS and W/W/W treatments. The treatment tissue concentrations ranged from 2.55 µM·g^-1^ FM to 7.65 µM·g^-1^ FM (**Table 4**).

cis-β-Ocimene concentrations were significantly influenced by season (F=48.61; *P ≤* 0.0001), lighting treatment (F=3.18; *P=*0.0004), and season*treatment interactions (F=1.81; *P=*0.0048) (**Table 2**). The June growing season had the highest tissue concentration, while the lowest concentration was found in September. Each of the growing seasons statistically separated. The season concentrations ranged from 145.81 µM·g^-1^ FM to 754.18 µM·g^-1^ FM (**Table 3**). The 6B/94R treatment again had the highest tissue concentrations, and statistically separated from some of the LED treatments as well as the HPS and W/W/W treatments. The NL control had the lowest concentration, but did not separate from the broadband treatments and many of the LED treatments. The treatment tissue concentrations ranged from 343.47 µM·g^-1^ FM to 756.25 µM·g^-1^ FM (**Table 4**).

Citronellyl Acetate concentrations were significantly influenced by season (F=13.31; *P ≤* 0.0001), lighting treatment (F=2.7; *P=*0.0024), and season*treatment interactions (F=1.75; *P=*0.0085) (**Table 2**). The June growing season had the highest tissue concentration, while the lowest concentration was found in January. September statistically separated from June and January, but not from the April growing season. The season concentrations ranged from 188.27 µM·g^-1^ FM to 193.66 µM·g^-1^ FM (**Table 3**). The 6B/94R treatment had the highest tissue concentrations, and statistically separated from some of the LED treatments, but not the NL control or broadband treatments. The high blue 470/450/420 treatment had the lowest concentration, but only separated from the 6B/94R treatment. The treatment tissue concentrations ranged from 189.24 µM·g^-1^ FM to 194.78 µM·g^-1^ FM (**Table 4**).

Linalool concentrations were significantly influenced by season (F=47.53; *P ≤* 0.0001), lighting treatment (F=2.17; *P=*0.0152), and season*treatment interactions (F=1.57; *P=*0.0262) (**Table 2**). The January growing season had the highest tissue concentration, while the lowest concentration was found in September. January, April, and June all statistically separated from September, but not from each other. The season concentrations ranged from 239.32 µM·g^-1^ FM to 925.12 µM·g^-1^ FM (**Table 3**). The 660/470/660 treatment had the highest tissue concentrations, but only statistically separated from the 660/420/660 treatment; none of the other treatments showed statistical separation. The treatment tissue concentrations ranged from 602.95 µM·g^-1^ FM to 924.86 µM·g^-1^ FM (**Table 4**).

trans-β-Ocimene concentrations were significantly influenced by season (F=80.44; *P ≤* 0.0001), lighting treatment (F=6.97; *P ≤* 0.0001), and season*treatment interactions (F=3.19; *P ≤* 0.0001) (**Table 2**). The June growing season had the highest tissue concentration, while the lowest concentration was found in September. September statistically separated from June and April, but not from the January growing season. The season concentrations ranged from 57.16 µM·g^-1^ FM to 351.14 µM·g^-1^ FM (**Table 3**). The 6B/94R treatment had the highest tissue concentrations, and statistically separated from all the other treatments except for 450/W/470. Many of the LED treatments do not show separation among themselves. The lowest concentration was found in the 660/420/660 treatment, but it did not separate from the NL control, many narrowband treatments, and the HPS and W/W/W treatments. The treatment tissue concentrations ranged from 111.37 µM·g^-1^ FM to 357.86 µM·g^-1^ FM (**Table 4**).

α-Ocimene concentrations were significantly influenced by season (F=195.03; *P ≤* 0.0001) and season*treatment interactions (F=2.00; *P=*0.0011), but not by treatment (F=1.29; *P=*0.2295) (**Table 2**). The September growing season had the highest tissue concentration, while the lowest concentration was found in April. September separated from the other seasons, but the January, April, and June seasons did not separate amongst themselves. September had approximately 5-20x tissue concentrations compared to other seasons. The season concentrations ranged from 75.23 µM·g^-1^ FM to 493.99 µM·g^-1^ FM (**Table 3**). Treatments did not statistically separate, and tissue concentrations ranged from 98.66 µM·g^-1^ FM to 212.20 µM·g^-1^ FM (**Table 4**).

β-Myrcene concentrations were significantly influenced by season (F=268.9; *P ≤* 0.0001), lighting treatment (F=5.15; *P ≤* 0.0001), and season*treatment interactions (F=2.83; *P ≤* 0.0001) (**Table 2**). The June growing season had the highest tissue concentration, while the lowest concentration was found in September. June did not statistically separate from April; September statistically separated from all other treatments and had drastically lower concentrations when compared to other seasons. The season concentrations ranged from 9.36 µM·g^-1^ FM to 407.65 µM·g^-1^ FM (**Table 3**). The 6B/94R treatment had the highest tissue concentrations, and statistically separated from the NL control, HPS, and W/W/W treatments. The 660/420/660 treatment had the lowest concentration, which separated from the 660/470/660, 450/W/470, and PhysioSpec treatments. The treatment tissue concentrations ranged from 203.27 µM·g^-1^ FM to 345.31 µM·g^-1^ FM (**Table 4**).

### Bicyclic monoterpenes

3-Caren-10-al concentrations were significantly influenced by season (F=17.21; *P ≤* 0.0001) and treatment (F=2.24; *P=*0.0123), but not by season*treatment interactions (F=1.06; *P=*0.3871) (**Table 2**). The September growing season had the highest concentration and was statistically separated from the lowest season, which was April. Season concentrations ranged from 1.21 µM·g^-1^ FM to 2.51 µM·g^-1^ FM (**Table 3**). The treatments 450/W/470 and 6B/94R were both significantly higher than the NL control, but the other treatments did not separate statistically. The treatment concentrations ranged from 1.32 µM·g^-1^ FM to 2.40 µM·g^-1^ FM (**Table 4**).

(+)-4-Carene concentrations were significantly influenced by season (F=27.75; *P ≤* 0.0001), lighting treatment (F=7.00; *P ≤* 0.0001), and season*treatment interactions (F=1.57; *P=*0.0254) (**Table 2**). The June growing season had the highest tissue concentration, while the lowest concentration was found in January. All of the season concentration averages statistically separated from each other. The season concentrations ranged from 16.93 µM·g^-1^ FM to 61.46 µM·g^-1^ FM (**Table 3**). The 6B/94R treatment had the highest tissue concentrations, and statistically separated from the NL control, HPS, and W/W/W treatments. The 660/420/660 treatment had the lowest concentration, which separated from the 660/470/660, 450/W/470, and 6B/94R treatments. The treatment tissue concentrations ranged from 18.67 µM·g^-1^ FM to 45.46 µM·g^-1^ FM (**Table 4**).

3-Carene concentrations were significantly influenced by season (F=24.28; *P ≤* 0.0001) and season*treatment interactions (F=2.35; *P=*0.0005), but not by lighting treatment (F=4.53; *P=*0.0521) (**Table 2**). The June growing season had the highest tissue concentration, but did not statistically separate from April or September. January growing season had the lowest concentration, separating from the other seasons. The season concentrations ranged from 379.21 µM·g^-1^ FM to 559.24 µM·g^-1^ FM (**Table 3**).

Camphene concentrations were significantly influenced by season (F=70.69; *P ≤* 0.0001), lighting treatment (F=9.41; *P ≤* 0.0001), and season*treatment interactions (F=1.52; *P=*0.0356) (**Table 2**). The September growing season had the highest tissue concentration, while the lowest concentration was found in January. The September season statistically separated from the other seasons, but January, April, and June did not separate amongst themselves. The season concentrations ranged from 23.41 µM·g^-1^ FM to 55.77 µM·g^-1^ FM (**Table 3**). The 450/W/470 treatment had the highest tissue concentrations, but did not statistically separate from the PhysioSpec and 660/470/660 treatments; the 450/W/470 did separate from the HPS, W/W/W, and NL control. The 660/420/660 treatment had the lowest concentration, which separated from the 660/470/660, 450/W/470, and PhysioSpec treatments. The treatment tissue concentrations ranged from 22.72 µM·g^-1^ FM to 51.23 µM·g^-1^ FM (**Table 4**).

Isoborneol concentrations were significantly influenced by season (F=22.21; *P ≤* 0.0001), lighting treatment (F=3.85; *P ≤* 0.0001), and season*treatment interactions (F=1.51; *P=*0.0367) (**Table 2**). The September growing season had the highest tissue concentration, while the lowest concentration was found in April. September, June, and April all statistically separated, but January did not separate from June and April. The season concentrations ranged from 23.53 µM·g^-1^ FM to 49.74 µM·g^-1^ FM (**Table 3**). The 6B/94R treatment had the highest tissue concentrations, and statistically separated from the NL control and HPS treatments, as well as some of the LED treatments. The 660/420/660 treatment had the lowest concentration, which did not separate from the NL control and the majority of LED treatments. The treatment tissue concentrations ranged from 22.05 µM·g^-1^ FM to 51.35 µM·g^-1^ FM (**Table 4**).

trans-Pinocarveol concentrations were significantly influenced by season (F=7.36; *P ≤* 0.0001), lighting treatment (F=3.32; *P=*0.0002), and season*treatment interactions (F=1.89; *P=*0.00027) (**Table 2**). The September growing season had the highest tissue concentration, while the lowest concentration was found in January. The season concentrations ranged from 3.60 µM·g^-1^ FM to 6.13 µM·g^-1^ FM (**Table 3**). The 6B/94R treatment again had the highest tissue concentrations, which separated from the 660/420/660, 660/450/660, and W/W/W treatments. The 660/420/660 treatment had the lowest concentration, but the only treatment that was statistically significantly different was the 6B/94R treatment. The treatment tissue concentrations ranged from 3.12 µM·g^-1^ FM to 7.30 µM·g^-1^ FM (**Table 4**).

trans-Sabinene hydrate concentrations were significantly influenced by season (F=158.38; *P ≤* 0.0001), lighting treatment (F=5.80; *P ≤* 0.0001), and season*treatment interactions (F=2.66; *P ≤* 0.0001) (**Table 2**). The June growing season had the highest tissue concentration, while the lowest concentration was found in January. September and January did not statistically separate. The season concentrations ranged from 10.04 µM·g^-1^ FM to 37.07 µM·g^-1^ FM (**Table 3**). The 660/470/660 treatment had the highest tissue concentrations, and statistically separated from the NL control, HPS, and W/W/W treatments. The 660/470/660 treatment did not separate from the 6B/94R treatment and some of the other LED treatments. The lowest concentration was found in the NL control, which did not separate from the HPS and W/W/W treatments. The treatment tissue concentrations ranged from 13.09 µM·g^-1^ FM to 25.92 µM·g^-1^ FM (**Table 4**).

α-Pinene concentrations were significantly influenced by season (F=73.74; *P ≤* 0.0001), lighting treatment (F=9.4; *P ≤* 0.0001), and season*treatment interactions (F=1.65; *P=*0.0152) (**Table 2**). The September growing season had the highest tissue concentration, while the lowest concentration was found in January. All of the season concentration averages statistically separated, except for April and June. The season concentrations ranged from 17.27 µM·g^-1^ FM to 46.27 µM·g^-1^ FM (**Table 3**). The 450/W/470 and 6B/94R treatments had the highest tissue concentrations and were not statistically separate. The 660/420/660 treatment again had the lowest concentration, which separated from the 660/470/660, 470/450/420, 450/W/470, and PhysioSpec treatments. The 660/420/660 treatment did not separate from the HPS, W/W/W, or the NL control. The treatment tissue concentrations ranged from 16.60 µM·g^-1^ FM to 41.75 µM·g^-1^ FM (**Table 4**).

β-Pinene concentrations were significantly influenced by season (F=49.46; *P ≤* 0.0001), lighting treatment (F=5.93; *P ≤* 0.0001), and season*treatment interactions (F=1.86; *P=*0.0032) (**Table 2**). β-Pinene followed a similar pattern to α-Pinene for both season and lighting treatment concentration. In addition, the ratio of α-Pinene to β-Pinene varied less than 8% across all treatments and seasons, and did not show any discernable pattern. The September growing season had the highest tissue concentration, while the lowest concentration was found in January. All of the season concentration averages statistically separated, except for April and June. The season concentrations ranged from 99.30 µM·g^-1^ FM to 208.12 µM·g^-1^ FM (**Table 3**). The 450/W/470 and 6B/94R treatments again had the highest tissue concentrations and were not statistically separate. The 660/420/660 treatment again had the lowest concentration, which separated from the 660/470/660, 470/450/420, 450/W/470, and 6B/94R treatments. The 660/420/660 treatment did not separate from the HPS, W/W/W, or the NL control. The treatment tissue concentrations ranged from 111.86 µM·g^-1^ FM to 190.27 µM·g^-1^ FM (**Table 4**).

### Cyclic monoterpenes

3-Menthene concentrations were significantly influenced by season (F=10.23; *P ≤* 0.0001), lighting treatment (F=4.23; *P ≤* 0.0001), and season*treatment interactions (F=2.34; *P ≤* 0.0001) (**Table 2**). The June growing season had the highest tissue concentration, while the lowest concentration was found in January. The January growing season separated from the other seasons, but April, June, and September did not separate amongst themselves. The season concentrations ranged from 24.22 µM·g^-1^ FM to 46.11 µM·g^-1^ FM (**Table 3**). The 12B/88R treatment had the highest tissue concentrations and separated from the HPS, and W/W/W, but not the NL control. The 660/420/660 treatment again had the lowest concentration, which separated from the 450/W/470 PhysioSpec treatments. The 660/420/660 treatment did not separate from the HPS, W/W/W, or the NL control. The treatment tissue concentrations ranged from 20.38 µM·g^-1^ FM to 53.10 µM·g^-1^ FM (**Table 4**).

d-Limonene concentrations were significantly influenced by season (F=29.8; *P ≤* 0.0001), lighting treatment (F=7.17; *P ≤* 0.0001), and season*treatment interactions (F=1.74; *P=*0.0076) (**Table 2**). The June growing season had the highest tissue concentration, while the lowest concentration was found in January. All of the season concentration averages statistically separated, except for April and September. The season concentrations ranged from 234.61 µM·g^-1^ FM to 387.51 µM·g^-1^ FM (**Table 3**). The 6B/94R treatment had the highest tissue concentration and was separated from the HPS, W/W/W, and NL control. The 660/420/660 treatment had the lowest concentration, which separated from the 660/470/660, 470/450/420, 450/W/470, and PhysioSpec treatments. The 660/420/660 treatment did not separate from the HPS, W/W/W, or the NL control. The treatment tissue concentrations ranged from 224.30 µM·g^-1^ FM to 401.22 µM·g^-1^ FM (**Table 4**).

Fenchyl acetate concentrations were significantly influenced by season (F=14.98; *P ≤* 0.0001), but not by lighting treatment (F=1.58; *P=*0.1019) or season*treatment interactions (F=0.62; *P=*0.9467) (**Table 2**). The April growing season had the highest tissue concentration, but it did not statistically separate from the January or June seasons. The lowest concentrations were found in the September season. The season concentrations ranged from 81.12 µM·g^-1^ FM to 149.80 µM·g^-1^ FM (**Table 3**). The treatment tissue concentrations did not statistically separate and ranged from 81.22 µM·g^-1^ FM to 115.00 µM·g^-1^ FM (**Tables 2**, **4**).

p-Menth-1-en-8-ol concentrations were significantly influenced by season (F=173.75; *P ≤* 0.0001), lighting treatment (F=10.61; *P ≤* 0.0001) and season*treatment interactions (F=2.56; *P ≤* 0.0001) (**Table 2**). The June growing season had the highest tissue concentration, while the lowest concentration was found in January. All of the season concentration averages statistically separated. The season concentrations ranged from 5.44 µM·g^-1^ FM to 19.69 µM·g^-1^ FM (**Table 3**). The 6B/94R treatment had the highest tissue concentration and was separated from the HPS, W/W/W, and NL control. The 660/420/660 treatment had the lowest concentration, which separated from the 660/470/660, 470/450/420, 450/W/470, and PhysioSpec treatments. The 660/420/660 treatment did not separate from the HPS or the NL control, as well as a few LED treatments. The treatment tissue concentrations ranged from 7.78 µM·g^-1^ FM to 17.03 µM·g^-1^ FM (**Table 4**).

### Sesquiterpenes

α-Humulene concentrations were significantly influenced by season (F=19.58; *P ≤* 0.0001) and lighting treatment (F=1.87; *P=*0.0420), but not by season*treatment interactions (F=1.12; *P=*0.3001) (**Table 2**). The September growing season had the highest tissue concentration, but did not separate from the June season. While the lowest concentration was found in January, it did not statistically separate from April. The season concentrations ranged from 53.69 µM·g^-1^ FM to 59.58 µM·g^-1^ FM (**Table 3**). The treatment tissue concentrations did not show statistical separation and ranged from 54.51 µM·g^-1^ FM to 59.41 µM·g^-1^ FM (**Tables 2**, **4**).

### Organosulfur

Isothiocyanatocyclopropane concentrations were significantly influenced by season (F=9.92; *P ≤* 0.0001), but not by lighting treatment (F=1.69; *P=*0.0752) or season*treatment interactions (F=0.85; *P=*0.7103) (**Table 2**). The September growing season had the highest tissue concentration, while the lowest concentration was found in April. The season concentrations ranged from 1.20 µM·g^-1^ FM to 2.28 µM·g^-1^ FM (**Table 3**). The treatment tissue concentrations did not show statistical separation, and ranged from 0.99 µM·g^-1^ FM to 2.32 µM·g^-1^ FM (**Table 4**).

2-Isobutylthiazole concentrations were significantly influenced by season (F=10.79; *P ≤* 0.0001), but not by lighting treatment (F=1.90; *P=*0.0591) or season*treatment interactions (F=1.23; *P=*0.2101) (**Table 2**). The September growing season had the highest tissue concentration, but did not statistically separate from the June season. The lowest concentration was found in January, but it did not separate from the April season. The season concentrations ranged from 5.57 µM·g^-1^ FM to 8.94 µM·g^-1^ FM (**Table 3**).

Dimethyl Sulfide concentrations were significantly influenced by season (F=25.15; *P ≤* 0.0001), lighting treatment (F=4.30; *P ≤* 0.0001), and season*treatment interactions (F=2.09; *P=*0.0005) (**Table 2**). The April growing season had the highest tissue concentration and statistically separated from all other seasons. The lowest season concentration was found in January, which also separated from all other seasons. The season concentrations ranged from 43.42 µM·g^-1^ FM to 121.98 µM·g^-1^ FM (**Table 3**). The 660/470/660 treatment had the highest tissue concentration and was separated from the W/W/W and NL control. The NL control had the lowest concentration, which separated from some of the narrowband treatments. The treatment tissue concentrations ranged from 32.68 µM·g^-1^ FM to 112.47 µM·g^-1^ FM (**Table 4**).

### Phenylpropanoids

Eugenol concentrations were significantly influenced by season (F=130.1; *P ≤* 0.0001), lighting treatment (F=9.94; *P ≤* 0.0001), and season*treatment interactions (F=4.43; *P ≤* 0.0001) (**Table 2**). The June growing season had the highest tissue concentration, while the lowest concentration was found in January. All of the season tissue concentration averages statistically separated. The season concentrations ranged from 43.16 µM·g^-1^ FM to 280.02 µM·g^-1^ FM (**Table 3**). The 6B/94R treatment had the highest tissue concentration and separated from all other treatments. The NL control had the lowest concentration, which separated from the 660/420/660, 660/450/660, 660/470/660, 470/450/420, 450/W/470, W/W/W, and PhysioSpec treatments. The treatment tissue concentrations ranged from 90.09 µM·g^-1^ FM to 283.16 µM·g^-1^ FM (**Table 4**).

Methyl Eugenol concentrations were significantly influenced by season (F=7.95; *P ≤* 0.0001), but not by lighting treatment (F=1.7; *P=*0.0997) or season*treatment interactions (F=1.5; *P=*0.1545) (**Table 2**). The September growing season had the highest tissue concentration, but did not statistically separate from the June season. The lowest concentration was found in January, but did not separate from April. The season concentrations ranged from 131.23 µM·g^-1^ FM to 172.11µM·g^-1^ FM (**Table 3**). The treatment tissue concentrations ranged from 158.11 µM·g^-1^ FM to 165.77 µM·g^-1^ FM (**Table 4**).

### Principal component analysis

**Figure 5:**
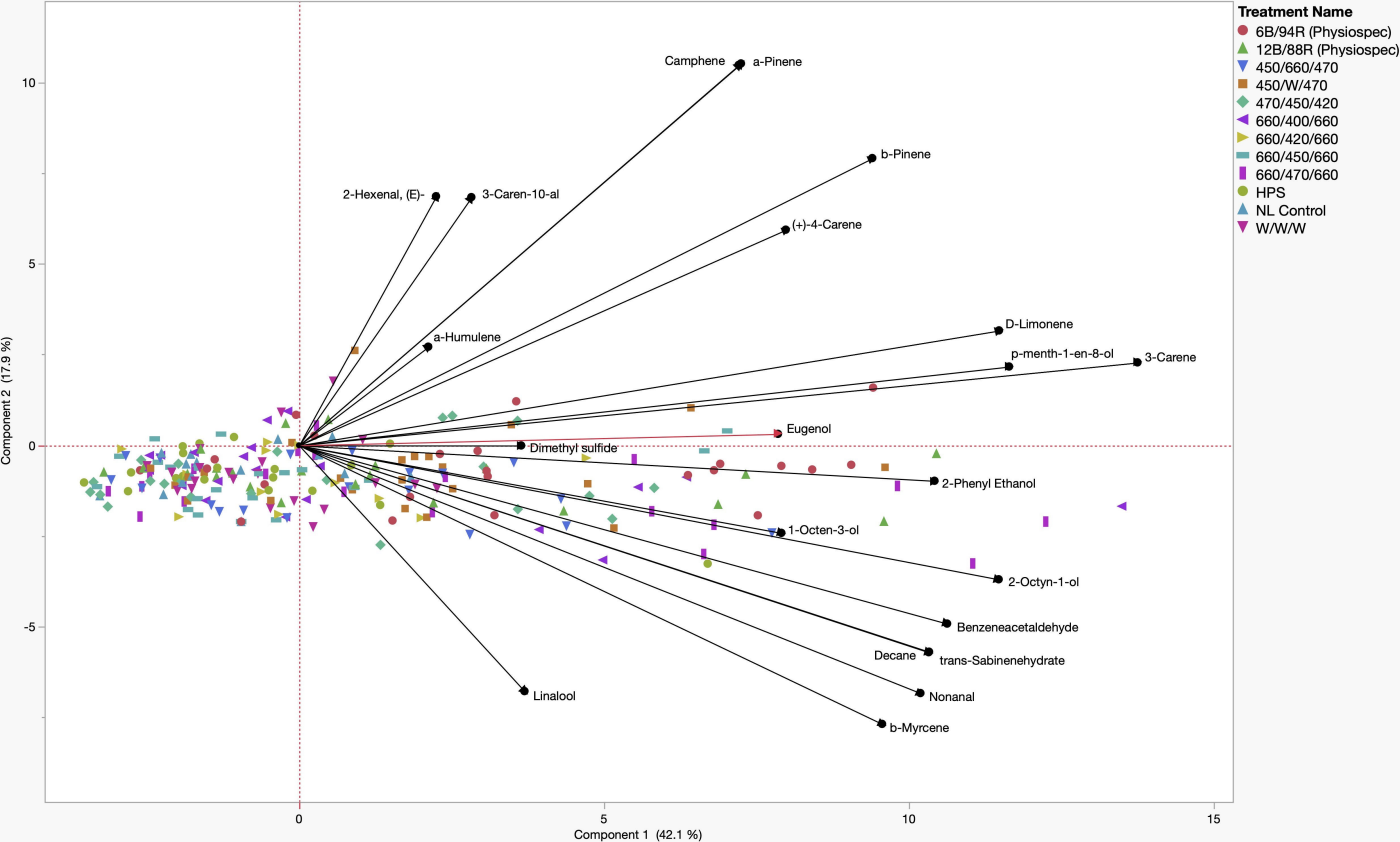
Principal Component Analysis (PCA) showing the biplot differentiation between sweet basil ‘Italian Large Leaf’ (*Ocimum basilicum* L.) aroma compound concentrations (black) grown under various supplemental lighting treatments.

A principal component analysis (PCA) comparison of key PVOCs and lighting treatments, represented using a biplot, revealed that two components with eigenvalues > 1 accounted for 60.0% of the total data variability (**Figure 5**). Component 1 accounted for 42.1% of the variability, while component 2 accounted for 17.9% of the data variability. Many key volatile compounds were positive discriminating factors for component 1 of varying magnitudes, while component 2 evenly separated compounds into positive and negative discriminating factors of various magnitudes. Quadrant I contained positive discriminating factors for both component 1 and component 2, which were the aroma compounds: (E)-2-Hexenal, α-Humulene, 3-Caren-10-al, Camphene, Linalool, α-Pinene, β-Pinene, d-Limonene, p-Menth-1-en-8-ol, 3-Carene, and Eugenol. Concentration increases of these compounds were generally associated with the narrowband LED treatments 470/450/420, 660/470/660, and 660/400/660. Quadrant II and Quadrant III do not contain any of the key aroma compounds and reveal that 450/660/470, 660/420/660, HPS, W/W/W, NL Control, and 660/450/660 are negatively associated with concentration increases of many key aroma volatiles, at various weights. Quadrant IV contained positive discriminating factors for component 1 and negative discriminating factors for component 2, which included the aroma compounds: 2-Phenyl Ethanol, 1-Octen-3-ol, 2-Octyn-1-ol, Benzeneacetaldehyde, trans-Sabinene hydrate, Nonanal, β-Myrcene, and Linalool. Concentration increases of these compounds were generally associated with the PhysioSpec (6B/94R, 12B/88R) and 450/W/470 treatments (**Figure 5**).

## Discussion

Light plays a critical role in the growth and development of many crops, including sweet basil. Spectral quality has a significant impact on secondary metabolism, which can directly influence the concentration of flavor and aroma compounds in plant tissues. Leveraging environmental controls and applying abiotic stressors has the ability to influence the secondary metabolism of high-value specialty crops. The results of this study demonstrate that spectral quality manipulations of supplemental greenhouse lighting can directly influence tissue concentrations of key aroma volatiles and other secondary metabolites in sweet basil. Total basil tissue VOC concentrations, in addition to many of the concentrations of specific volatile compounds, were significantly impacted by the growing season and lighting treatment.

In **Figure 3**, total VOC concentrations are displayed based on lighting treatment and have been broken down into their respective compound classes. All of the SL treatments provide the same intensity (100 µmol^**.**^m^-2.^s^-1^) for the same duration (24 h); this isolates SL spectral quality as a primary independent variable, while the non-supplemented NL control provides a baseline to compare treatments. Statistical analysis reveals that total VOC concentrations separated across lighting treatments, and a few general patterns emerge.

First, the 6B/94R treatment had the highest total VOC concentration, but did not statistically separate from the 12B/88R treatment. The total VOC concentration of the 450/660/470 (i.e., 2B:1R or 66.6B/33.3R) was significantly lower than the 6B/94R treatment, but did not separate from the 12B/88R treatment. In one recent study with green basil (*Ocimum basilicum* L.), the chemical composition of essential oil and total phenolic content was improved by growing plants under 30B/70R light when compared to monochromatic, dichromatic, and broadband sources (Hosseini et al., 2018). Pennisi et al. also found that a ratio of 3 R:B (i.e., 1B:3R) was ideal for resource use efficiency and flavor volatile production in basil (*Ocimum basilicum* L.) (Pennisi et al., 2019a). Our group determined that for ‘Genovese’ sweet basil (*Ocimum basilicum* L.), maximum concentrations for key compounds varied among narrowband lighting treatment, but most monoterpenes and diterpenes evaluated were highest under a SL treatment of 20B/80R to 50B/50R (Hammock et al., 2021). When comparing previous studies, it is clear that light-mediated responses (i.e., isoprenoid and phenylpropanoid metabolism changes based on B:R ratio) are species and even variety-specific (Taulavuori et al., 2016b; Toscano et al., 2021). Based on the results of this experiment and current literature, we recommend supplemental narrowband B:R ratios between 20B/80R and 5B/95R (i.e., 1B:4R to 1B:20R) with the intensity of 100-200 µmol^**.**^m^-2.^s^-1^ for 12-24 h daily for high-value specialty crops under standard greenhouse conditions, with direct consideration of the unique ambient spectra and DLI provided for any given location and growing season. Further studies should also be conducted on different varieties of basil and other high-value specialty crop species to determine ideal supplemental B:R ratios.

Second, the high blue treatment (470/450/420) performed as well as the 2B:1R narrowband treatment (450/660/470). It has generally been shown that high intensities of blue wavelengths promote the synthesis of many phenols and terpenoids (Colquhoun et al., 2013; Carvalho et al., 2016; Taulavuori et al., 2016b; Toscano et al., 2021; Kivimaenpa et al., 2022). That being said, it is likely that the ambient solar spectrum during the natural daylight hours was sufficient to provide additional wavelengths necessary for normal physiological function and secondary metabolite concentrations as observed in this experiment. Some studies have shown that monochromatic or dichromatic sole-source lighting can be detrimental to primary and secondary metabolic function (Carvalho et al., 2016; Jishi et al., 2016; Kong et al., 2019). Adding small amounts of discrete wavelengths relative to the total intensity of the ambient spectrum has potential to impart desirable secondary metabolic effects while minimizing electrical energy use.

Third, the total VOC concentrations of the four 1B:2R treatments (660/400/660, 660/420/660, 660/450/660, and 660/470/660) each statistically separated from each other. The 660/470/660 had the highest total concentrations, while the 660/420/660 had the lowest. This demonstrates the impact of discrete narrowband blue wavelengths (with the same spectra/intensity of red wavelengths) on total VOC concentration. The 660/470/660 treatment was the only 1B:2R treatment to statistically separate from the NL control. A sole-source lighting study found that several quality parameters and secondary metabolite concentrations in basil (*Ocimum basilicum* L.) and strawberry (*Fragaria x ananassa*) were improved by using a 0.7 R:B ratio (i.e., 1.4B:1R) (Piovene et al., 2015). A study comparing natural light under standard greenhouse conditions to indoor sole-source lighting treatments determined significant increases to monoterpene concentrations when using blue/red/yellow and blue/red/green in growth chambers (Carvalho et al., 2016). A similar indoor lighting study found that using broadband sources with higher intensities of blue wavelengths (i.e., 1B:2.5R vs. 1B:4R) influenced terpenoid and phenylpropanoid concentrations in relation to phenolic acids (Kivimaenpa et al., 2022). This further demonstrates the wide range of effects from sole source, and SL can be species and variety-specific.

This study differs from many previous studies evaluating basil aroma volatiles in that sole-source lighting is utilized as compared to supplementing the natural solar spectrum with specific wavebands. The ambient solar spectra, as well as differing species-specific light mediated responses it imparts, adds an additional layer of complexity. Determining the influence of varying light spectra with and without ambient solar spectra, as well as comparing both, will improve our understanding of plant/light interaction as well as help commercial growers in both indoor farm and greenhouse operations.

Based on this comparison of discrete blue wavelengths, if using supplemental narrowband B/R lighting for sweet basil production, we also recommend 450 nm blue additions to 660 nm red (± 20 nm) for ideal total VOC bioaccumulation in basil, using the aforementioned B:R ratio range (i.e., 1B:4R to 1B:20R). Further evaluation is warranted on 420 nm in relation to VOC profiles. The wavelengths around 420 nm have been shown to promote VOCs and other secondary metabolites (Singh et al., 2015; Hasan et al., 2017; Ueda et al., 2021). The 660/420/660 actually produced the lowest total VOC concentration, and was statistically lower than many of the narrowband treatments. Further, it did not statistically separate from the NL control. It is likely that the 420 nm wavelengths promoted the production of other non-volatile secondary metabolites which are not detectable using HS GC-MS, such as carotenoids and flavonoids. Additional experiments evaluating the entire secondary metabolome with various analytical techniques in addition to metabolomics and/or transcriptomics would further elucidate the specific regulation of key pathways. It is also pertinent for future studies to incorporate both primary and secondary metabolic data to determine further resource allocation based on light responses.

Fourth, the broadband spectrum lighting treatments had mixed performance in terms of total VOC concentration. The HPS and W/W/W treatments did not statistically separate from the NL control. The high-blue broad-spectrum treatment (450/W/470) actually had the second-highest total VOC concentration, and did not statistically separate from the 6B/94R and 12B/88R treatments. Further exploration into specific intensities of discrete narrowband wavelengths within broad-spectrum supplement lighting, specifically blue wavelengths, is needed. Additionally, the color temperature (i.e., Kelvin) of broadband white light, should be evaluated in terms of secondary metabolic resource allocation.

**Figure 4** shows total VOC concentrations based on the growing season and have been broken down into their respective compound classes. All of the total VOC concentrations statistically separated across the growing season. In the order of highest to lowest total VOC concentrations, June had the highest total VOC concentration (6140 µM·g^-1^ FM), April had the second highest total VOC concentration (5015 µM·g^-1^ FM), January had the third highest total VOC concentration (3840 µM·g^-1^ FM), and finally, September had the lowest concentration (2760 µM·g^-1^ FM). Greenhouse growing conditions and environmental parameters were similar for all four growing seasons in this experiment (**Table 1**). The non-supplemented NL control provides a baseline to compare across all seasons. Greenhouse day and night temperatures were held constant within 2 °C across all growing seasons, which isolates two of the primary independent variables utilized in this experiment: change in spectral quality from ambient sunlight across growing seasons and change in total DLI from ambient sunlight across growing seasons. It would generally be expected that increasing the DLI from ambient sunlight would indirectly increase total volatile concentration, due to the increased primary metabolic capacity and ability to allocate additional resources to secondary pathways (Faust et al., 2005; Garland et al., 2010; Currey and Lopez, 2015). That being said, it was interesting to see that September had the second-highest DLI, but the lowest total VOC concentration. Additionally, January had the lowest DLI, but had the third-highest total VOC concentration. June had the highest DLI, as well as the highest total VOC concentration (**Table 4**). This indicates that the change in both total DLI and spectral quality of the ambient sunlight across growing seasons directly influences total VOC concentrations.

The June growing seasons generally had the highest tissue concentrations of specific volatile compounds, while January generally had the lowest concentrations. Many of the compounds followed the trend of higher tissue concentrations as the average total growing season DLI increased. That being said, some notable exceptions do not trend with seasonal DLI, demonstrating the complex interaction between seasonal spectral quality and secondary metabolism. These exceptions can be separated into three groups. First, the compounds 2-Octyn-1-ol, Nonanal, cis-β-Ocimene, Linalool, and β-Myrcene had the lowest concentrations in the September growing season rather than the January growing season. Second, the compounds 3-Caren-10-al, Camphene, Isoborneol, α-Pinene, and β-Pinene all had the highest concentrations in the September growing season rather than June growing season. Third, Dimethyl Sulfide was the only compound in this experiment to have the highest tissue concentration in the April growing season, rather than the June growing season. It was interesting to observe the various effects on compounds across different classes and metabolic pathways, which warrants further exploration using analytical techniques as well as metabolomics.

For many of the statistically significant compounds evaluated in this study, the 6B/94R treatment produced the highest concentrations, while the lowest was generally observed in the 660/420/660 treatment. 16 VOCs were determined to be responsible for approximately 90% of the total response area as well as variations in the aromatic profile, which is in agreement with a similar experiment (Pennisi et al., 2019a). Four compounds deviated from this pattern, all of which have proven significant in sensory perception for sweet basil.

Nonanal has a strong and unmistakable scent. Its aroma is described as fatty, citrusy orange peel with notes of sweet floral and rose petals. It adds warmth to fragrances, boosts freshness in floral compositions, and offers a distinctive aldehydic odor. Nonanal rounds off the smell of perfumes and helps make them more palatable to the nose. It also has the ability to lend its waxy character to other flavors, giving it a unique sensorial appeal (Sell, 2019). The highest tissue concentration of Nonanal was found under the 660/470/660 treatment, which was more than double the concentration found in the NL control.

Linalool is a naturally occurring terpene found in many flowers, spices, and other higher plants. The aroma of linalool is described as sweet, fruity, floral, and herbaceous. It has citrus and spice notes, with a light woodsy character reminiscent of lavender and bergamot oil. Linalool is commonly found in herbs such as oregano, thyme, marjoram, rosemary, and basil (Meligaard et al., 2007; Sell, 2019). Linalool tissue concentrations were highest in the 660/470/660, while the lowest was under the 660/420/660 treatment; these were the only two treatments that statistically separated for Linalool.

Dimethyl Sulfide has a strong, pungent aroma that can be described as earthy, fishy, or sulfuric. It is often compared to the smell of cooked cabbage and is used as an ingredient in certain types of food flavorings. It has high bioactivity and can be detected in extremely small concentrations, even at concentrations below 0.1 ppm; relatively low concentrations can lead to negative sensory perception in humans (Meligaard et al., 2007; Sell, 2019). Interestingly, the highest concentration was found in the 660/470/660, but was only statistically separate from the W/W/W and the NL control. It should be noted that the addition of narrowband LED treatments directly increased the concentration of this negatively perceived compound, while the same intensity of broad-spectrum white light (W/W/W) was statistically lower. The broad spectrum white and the NL control, despite different DLIs, did not statistically separate, suggesting that spectral quality has a greater influence on Dimethyl Sulfide tissue concentrations than DLI. Other sulfur compounds in this experiment did not show statistically significant differences in concentration across lighting treatments.

Eugenol is a phenylpropanoid with a sweet, spicy, nutty, and woody aroma, with notes of clove, cinnamon, and allspice. Its scent is reminiscent of bay leaf and is often used as a food flavoring. Eugenol is known for its antimicrobial properties and therapeutic properties. It has been used to create unique perfumes, adding depth and complexity to scents, especially those with a floral or citrus character. It is commonly used to flavor commercially produced food products (Lawless, 2010; Sell, 2019). The highest tissue concentrations were found under the 6B/94R treatment, while the lowest concentrations were found under the NL control, an almost 3.5x difference. The 660/420/660 treatment did not statistically separate from any of the other LED treatments or the NL controls. Additionally, Methyl Eugenol, the synthesis of which is directly related to Eugenol, was not statistically significant across lighting treatments.

Four terpenoid compounds of key aroma volatiles, consisting of different chemical classes at various points within the isoprenoid pathway, followed the same general pattern across lighting treatments. These include α-Pinene, β-Pinene, Camphene, and d-Limonene.

α-Pinene and β-Pinene are two isomers found ubiquitously in nature, most notably in the essential oils of coniferous trees. α-pinene has a sharp, piney aroma. β-pinene, on the other hand, has an earthy and herbaceous scent reminiscent of rosemary and sage. The main difference between these two terpenes is their perceived intensity, as α-Pinene’s sharp and intense aroma stands out from β-pinene’s more subtle earthy notes. Both terpenes have significant culinary and therapeutic potential (Sell, 2019).

Camphene has a strong, piney, camphoraceous aroma with hints of fir and spices. It is often described as having citrus notes with an underlying musky sweetness. Camphene is used in perfume creation to add a woody and earthy edge to fragrances, particularly those with fresh and herbal elements. Camphene accentuates other flavors like citrus, mint, and earthy notes in food flavorings (Lawless, 2010; Sell, 2019).

d-Limonene has a strong and distinct citrus scent reminiscent of oranges and lemons. It is often described as having a distinctive sweet orange aroma, with notes of lemon and lime. d-Limonene is used in the production of perfumes, cosmetics, food flavorings, and cleaning products due to its pleasant smell. Despite being an undernote of basil aroma, it still plays a significant role in the overall aroma perception of some sweet basil varieties (Lawless, 2010; Sell, 2019).

These four terpenoid compounds all have various physical and chemical properties and reside at different locations within the isoprenoid pathway, but generally maintain the same pattern across lighting treatments. This suggests that these pathways are receiving more upstream products to the biosynthesis of some terpenoid compounds (i.e., resource allocation is being shifted to terpenoid pathways due to light stress induced from discrete combinations of narrowband wavelengths), while other specific compounds within the isoprenoid and phenylpropanoid pathway are being differentially regulated based on varying spectral quality and/or DLIs.

Although basil is one of the most popular herbs globally, it can be challenging to characterize in terms of light-mediated sensory properties at the genomic and phenotypic levels. One limitation to evaluating the interaction of light and sensory quality is that there are over sixty varieties of *Ocimum basilicum*, each with specific light-mediated secondary metabolic responses; this complicates the comparison of studies found in current literature (Blank et al., 2004). These light-mediated responses cannot be easily generalized to other high-value specialty crops, which provides numerous unique research opportunities.

Headspace GC-MS is a commonly used analytical technique used to qualify and quantify VOCs from many types of biological samples. It is particularly useful for metabolomics and characterizing aroma profiles for plant tissues and food products. In this experiment, all volatile concentration units are reported in µM·g^-1^ FM. This unit (compared to µmol·g^-1^ FM) was utilized because it incorporates the concentration of each analyte per unit volume of headspace gas above the plant tissue (i.e., samples the dynamic and complex gaseous matrix which contains numerous pertinent VOCs), which is important for sensory-based studies. Further, VOCs are highly localized and typically require other GC-MS techniques (i.e., liquid solvent extraction/injection) to accurately quantify in terms of µmol·g^-1^ of homogenized plant tissue. HS analysis is an ideal GC-MS sampling technique involving sensory studies with plant flavor/aroma, because it accounts for the dynamic release of volatiles from plant tissues under repeatable conditions and can be incorporated with olfactometry (GC-O). Humans primarily detect aroma volatiles that have been volatilized (i.e., released from trichomes and other specialized structures), which then induces an olfactory response. By accurately calibrating analytical standards to known headspace concentrations, we can determine the micro molarity of each analyte, within a known volume of dynamic gaseous headspace matrix, under repeatable conditions (i.e., temperature and pressure using inert analytical grade He) to which a consumer would be exposed to during consumption. Future studies should use a variety of analytical as well as molecular techniques to determine the primary and secondary metabolic impacts of spectral quality. A multidisciplinary approach would allow for greater insight into the complex interactions between light and plant physiology. This could result in the development of light recipe guidelines based on location and weather conditions, to dynamically attenuate SL spectra to the natural solar spectra as it changes across seasons; season-specific lighting regimes have the potential to maintain consistent flavor and aroma quality throughout the year.

This experiment utilized continuous low-intensity light supplements, which have the potential to manipulate secondary metabolite bioaccumulation while efficiently increasing crop DLI (i.e., utilizing cheaper off-peak electrical rates during night hours). Because basil is grown to vegetative maturity and does not require a photoperiodic response, it tolerates 24 h SL very well; other crops will experience deleterious effects when using this type of lighting regime. Increasing the intensity and/or manipulating the duration of SL also has the potential to differentially influence secondary metabolic profiles and should be further evaluated under greenhouse and growth chamber conditions.

While narrowband B/R wavelengths have been shown to increase total and specific VOC concentrations, varying levels of impact have been observed among different species and specific SL spectral qualities; further exploration into discrete narrowband wavelengths at varying ratios is warranted (i.e., +/- 2 nm within the ambient spectrum). This could be used to push certain secondary metabolic pathways that could be used to improve flavor, the concentration of phytonutrients, human health benefits, and marketability. Many studies have demonstrated the species-specific nature of secondary metabolic light-mediated responses, which provides a vast range of research opportunities. Factors such as yield, nutrition content, phytonutrient concentrations, texture, and visual characteristics should also be considered when selecting SL regimes. Certain niche markets may utilize the findings of this study to improve flavor quality or push certain metabolic pathways (i.e., terpenoid and phenylpropanoid).

This study utilizes free sunlight and is intended to inform greenhouse basil production with SL requirements. It would be valuable from a scientific perspective to further explore similar methodologies, analytical techniques, and narrowband wavelengths in growth chambers with sole source lighting to determine the influence of ambient solar spectra and if similar results occur without the ambient solar being present. The results of this study show the merit of supplementing broad-spectrum ambient sunlight with targeted discrete narrowband wavelengths to manipulate secondary metabolism. Utilizing growth chambers to manipulate photoperiods and DLI of distinct spectral quality supplements would also prove beneficial for growers, as it would eliminate potential confounding factors from variation in ambient sunlight (i.e., weather, growing location, etc.), as well as provide comparative data for operations that rely on sole-source lighting, such as indoor farms and other types of controlled environment agriculture operations.

The results of this experiment will provide useful information on how SL can be used to optimize the sensory characteristics of sweet basil and provide a baseline to explore other high-value specialty crops. It is possible to significantly alter secondary metabolism by using sole source narrowband lighting, as well as narrowband supplements to ambient sunlight. Further research is required to determine patterns within different specialty crops and how wavelengths can be optimized for daily and seasonal changes in the solar spectrum.

## Conclusions

In this study, we explored, identified, and quantified several important volatile organic compounds with known influence on sensory perception and/or plant physiological processes of greenhouse-grown sweet basil. We determined that the spectral quality from SL sources, in addition to changes in the spectral quality and DLI of ambient sunlight across growing seasons, directly influence aroma volatile concentrations. Further, we found that specific ratios of narrowband blue/red wavelengths, combinations of discrete narrowband wavelengths, and broadband wavelengths directly influence the basil aroma profile as well as specific compounds. The results also show that variation in spectral quality and DLI across seasons can dramatically influence aroma concentrations. Narrowband treatments generally produced higher VOC compound concentrations; based on the results of this experiment and current literature, we suggest supplemental narrowband wavelengths blue (450-470 nm) and red (660-700 nm) at a ratio of approximately 10B/90R at 100-200 µmol^**.**^m^-2.^s^-1^ for 12-24 h for maximum total VOC concentration and key individual aroma volatile concentrations in greenhouse-grown ‘Italian Large Leaf’ sweet basil; future experiments should determine the influence of lighting regimes on VOC profiles in relation to consumer perception and preference. This experiment demonstrates the ability to use discrete narrowband wavelengths to augment the natural solar spectrum in order to provide an optimal light environment across growing seasons. Further, narrowband SL can be used to manipulate key flavor and aroma compound concentrations, which can directly impact human sensory perception. Future work should incorporate different analytical and metabolic techniques to determine the impact on important aroma volatiles as well as other primary and secondary metabolites; this includes any potential species-specific effects that spectral quality may have on plant physiology with the potential to indirectly impact sensory quality through light-mediated metabolic resource allocation.

## Data availability statement

The raw data supporting the conclusions of this article will be made available by the authors, without undue reservation.

## Author contributions

Conceptualization – HH and CS. Methodology – HH and CS. Software – HH and CS. Validation – HH. Formal analysis – HH. Investigation – HH. Resources – CS. Data curation – HH. Writing (original draft preparation) - HH. Writing (review and editing) – HH and CS. Visualization – HH. Supervision and project administration – CS. Funding acquisition – CS. All authors contributed to the article and approved the submitted version.
